# Pycallingcards: an integrated environment for visualizing, analyzing, and interpreting Calling Cards data

**DOI:** 10.1093/bioinformatics/btae070

**Published:** 2024-02-07

**Authors:** Juanru Guo, Wenjin Zhang, Xuhua Chen, Allen Yen, Lucy Chen, Christian A Shively, Daofeng Li, Ting Wang, Joseph D Dougherty, Robi D Mitra

**Affiliations:** Department of Genetics, Washington University in St. Louis School of Medicine, Saint Louis, MO 63110, United States; Edison Family Center for Genome Sciences and Systems Biology, Washington University in St. Louis School of Medicine, Saint Louis, MO 63110, United States; Department of Genetics, Washington University in St. Louis School of Medicine, Saint Louis, MO 63110, United States; Edison Family Center for Genome Sciences and Systems Biology, Washington University in St. Louis School of Medicine, Saint Louis, MO 63110, United States; Department of Genetics, Washington University in St. Louis School of Medicine, Saint Louis, MO 63110, United States; Edison Family Center for Genome Sciences and Systems Biology, Washington University in St. Louis School of Medicine, Saint Louis, MO 63110, United States; Department of Genetics, Washington University in St. Louis School of Medicine, Saint Louis, MO 63110, United States; Department of Psychiatry, Washington University in St. Louis School of Medicine, Saint Louis, MO 63110, United States; Department of Genetics, Washington University in St. Louis School of Medicine, Saint Louis, MO 63110, United States; Edison Family Center for Genome Sciences and Systems Biology, Washington University in St. Louis School of Medicine, Saint Louis, MO 63110, United States; Department of Genetics, Washington University in St. Louis School of Medicine, Saint Louis, MO 63110, United States; Edison Family Center for Genome Sciences and Systems Biology, Washington University in St. Louis School of Medicine, Saint Louis, MO 63110, United States; Department of Genetics, Washington University in St. Louis School of Medicine, Saint Louis, MO 63110, United States; Edison Family Center for Genome Sciences and Systems Biology, Washington University in St. Louis School of Medicine, Saint Louis, MO 63110, United States; Department of Genetics, Washington University in St. Louis School of Medicine, Saint Louis, MO 63110, United States; Edison Family Center for Genome Sciences and Systems Biology, Washington University in St. Louis School of Medicine, Saint Louis, MO 63110, United States; McDonnell Genome Institute, , Washington University in St. Louis School of Medicine, Saint Louis, MO, 63110, United States; Department of Genetics, Washington University in St. Louis School of Medicine, Saint Louis, MO 63110, United States; Department of Psychiatry, Washington University in St. Louis School of Medicine, Saint Louis, MO 63110, United States; Intellectual and Developmental Disabilities Research Center, Washington University School of Medicine, Saint Louis, MO 63108, United States; Department of Genetics, Washington University in St. Louis School of Medicine, Saint Louis, MO 63110, United States; Edison Family Center for Genome Sciences and Systems Biology, Washington University in St. Louis School of Medicine, Saint Louis, MO 63110, United States; McDonnell Genome Institute, , Washington University in St. Louis School of Medicine, Saint Louis, MO, 63110, United States; Intellectual and Developmental Disabilities Research Center, Washington University School of Medicine, Saint Louis, MO 63108, United States

## Abstract

**Motivation:**

Unraveling the transcriptional programs that control how cells divide, differentiate, and respond to their environments requires a precise understanding of transcription factors’ (TFs) DNA-binding activities. Calling cards (CC) technology uses transposons to capture transient TF binding events at one instant in time and then read them out at a later time. This methodology can also be used to simultaneously measure TF binding and mRNA expression from single-cell CC and to record and integrate TF binding events across time in any cell type of interest without the need for purification. Despite these advantages, there has been a lack of dedicated bioinformatics tools for the detailed analysis of CC data.

**Results:**

We introduce Pycallingcards, a comprehensive Python module specifically designed for the analysis of single-cell and bulk CC data across multiple species. Pycallingcards introduces two innovative peak callers, CCcaller and MACCs, enhancing the accuracy and speed of pinpointing TF binding sites from CC data. Pycallingcards offers a fully integrated environment for data visualization, motif finding, and comparative analysis with RNA-seq and ChIP-seq datasets. To illustrate its practical application, we have reanalyzed previously published mouse cortex and glioblastoma datasets. This analysis revealed novel cell-type-specific binding sites and potential sex-linked TF regulators, furthering our understanding of TF binding and gene expression relationships. Thus, Pycallingcards, with its user-friendly design and seamless interface with the Python data science ecosystem, stands as a critical tool for advancing the analysis of TF functions via CC data.

**Availability and implementation:**

Pycallingcards can be accessed on the GitHub repository: https://github.com/The-Mitra-Lab/pycallingcards.

## 1 Introduction

Cells divide, differentiate, and respond to their environments by modulating the expression of their genes. Such transcriptional changes are orchestrated by transcription factors (TFs), which bind to regulatory DNA sequences and recruit chromatin remodelers, RNA polymerase II holoenzyme, and other general TFs. Due to their central role in organizing gene expression, TFs are the subject of intense study and there are many outstanding questions about the mechanisms they use to enact gene expression programs (Zeitlinger 2020). Since TFs act by binding DNA, either directly or indirectly, efforts to answer these questions are greatly aided by methods such as ChIP-Seq (Park 2009), DamID (Greil *et al.* 2006), ChIP-exo (Rossi *et al.* 2018), Cut-and-Run (Baas *et al.* 2005), Cut-and-Tag (Kaya-Okur *et al.* 2019), and Transposon Calling Cards (CC) (Wang *et al.* 2007, 2011, 2012, Liu *et al.* 2020, Kfoury *et al.* 2021) that are used to map the genome-wide binding of a TF of interest.

The CC method is unique in that it can record binding events at one time point that can be read out at a later time (Cammack *et al.* 2020). In the CC method, a TF is fused to a transposase (most often PBase), and the transposase-TF fusion inserts transposons in the genome proximal to where the TF binds, leaving the transposon as a “Calling Card” marking the transient binding of the TF. The preferred type of transposon used in the CC method is an engineered PBase transposon called a self-reporting transposon (SRT). SRTs carry a ubiquitous promoter that drive the transcription of a reporter gene with no poly-A termination signal, so that the transcript continues into the adjacent genome, allowing transposon insertions to be read out via RNA sequencing. This reaction is compatible with single-cell platforms, and thus single-cell Calling Cards (scCC) (Moudgil *et al.* 2020), enable the readout of TF binding events and RNA-gene expression from tens of thousands of individual cells in parallel. This permits the analysis of TF function in heterogeneous tissues. Finally, if the transposase is not fused to a TF it has a natural affinity for the BRD4 chromatin modifier which is associated with enhancers and superenhancers (Gogol-Döring *et al.* 2016). Thus, a “default” CC experiment records enhancer utilization.

Most methods that measure TF binding in bulk samples (Zhang *et al.* 2008, Meers *et al.* 2019) as well as single-cell methods that measure RNA-expression or chromatin accessibility (Satija *et al.* 2015, Stegle *et al.* 2015, Wolf *et al.* 2018, Eraslan *et al.* 2019, Granja *et al.* 2021) have a number of associated bioinformatics tools to aid in the analysis of the data generated by these methods. Heretofore, there were no equivalent packages for the comprehensive analysis of CC data. Previously, Python modules such as ccf_tools (for python2) (Kfoury *et al.* 2021) and Blockify (Moudgil *et al.* 2020) were utilized to call CC peaks, but neither are user-friendly, nor do they provide an integrated environment for visualization, comparison with orthogonal RNA-seq and ChIP-seq datasets, or motif finding. Because CC data are substantially different from other ’omics data such as RNA-seq, ChIP-seq, and ATAC-seq, it is usually not appropriate to use the existing packages for those methods to analyze CC data as many of the statistical assumptions used in these packages are not valid for CC data (Park 2009, Saliba *et al.* 2014, Buenrostro *et al.* 2015, Kolodziejczyk *et al.* 2015). As a result, there is a critical need for a dedicated package that integrates peak-calling, visualization, and the downstream analysis of CC and scCC data to facilitate investigation into TF function.

In this paper, we present Pycallingcards, a Python (Sanner *et al.* 1999) module to analyze both scCC and bulk CC data in human, mouse, and yeast. This package introduces two new peak callers, CCcaller and Model-based Analysis for Calling Cards (MACCs), which improve the accuracy and speed of identifying TF binding sites from CC data. In order to benchmark these peak callers, we mapped 20 million PBase transposon insertions (Brd4 directed), the largest such dataset generated to date. Our analysis shows that CCcaller and MACCs are more accurate and efficient than previous peak callers. Pycallingcards also contains many new functions that allow for the integrative analysis of CC data and RNA-seq data to discover the relationship between TF binding and gene expression. Pycallingcards is designed for use in Jupyter notebooks, and this interface allows for rapid data visualization and exploration. The package can also help researchers compare CC data with other relevant genomic datasets in order to better understand the functional consequences of TF binding, and it is designed to be accessible to researchers with even a limited computational background. Finally, Pycallingcards can interface with the Python data science ecosystem to provide a scalable and extensible framework for the development of new CC data-related and machine learning methods.

To highlight the utility of Pycallingcards, we used it to reanalyze two published datasets: scCC Mouse cortex data (Moudgil *et al.* 2020) and bulk glioblastoma (GBM) CC data collected in female and male samples (Kfoury *et al.* 2021). The mouse cortex data both maps the binding of Brd4 and measures the gene expression in each cell, in parallel. In this study, we reveal cell-type specific binding sites and identify potential mechanisms by which Brd4 may regulate development. The GBM data were collected to provide insights into the epigenentic basis of sex differences in GBM by mapping Brd4 binding in an isogenic murine model comprised male and female astrocytes that were transformed via a combined loss of neurofibromin and p53 function (Kfoury *et al.* 2021). Using Pycallingcards, we found previously unidentified TF regulators of the transcriptional differences between sexes, thus providing new insights for future study and treatment. These analyses form the basis of a detailed and user-friendly Jupyter notebook-based tutorial available at https://pycallingcards.readthedocs.io/en/latest/ to facilitate the adoption of this package.

## 2 Results

### 2.1 Overview of Pycallingcards workflow

**Figure 1.**
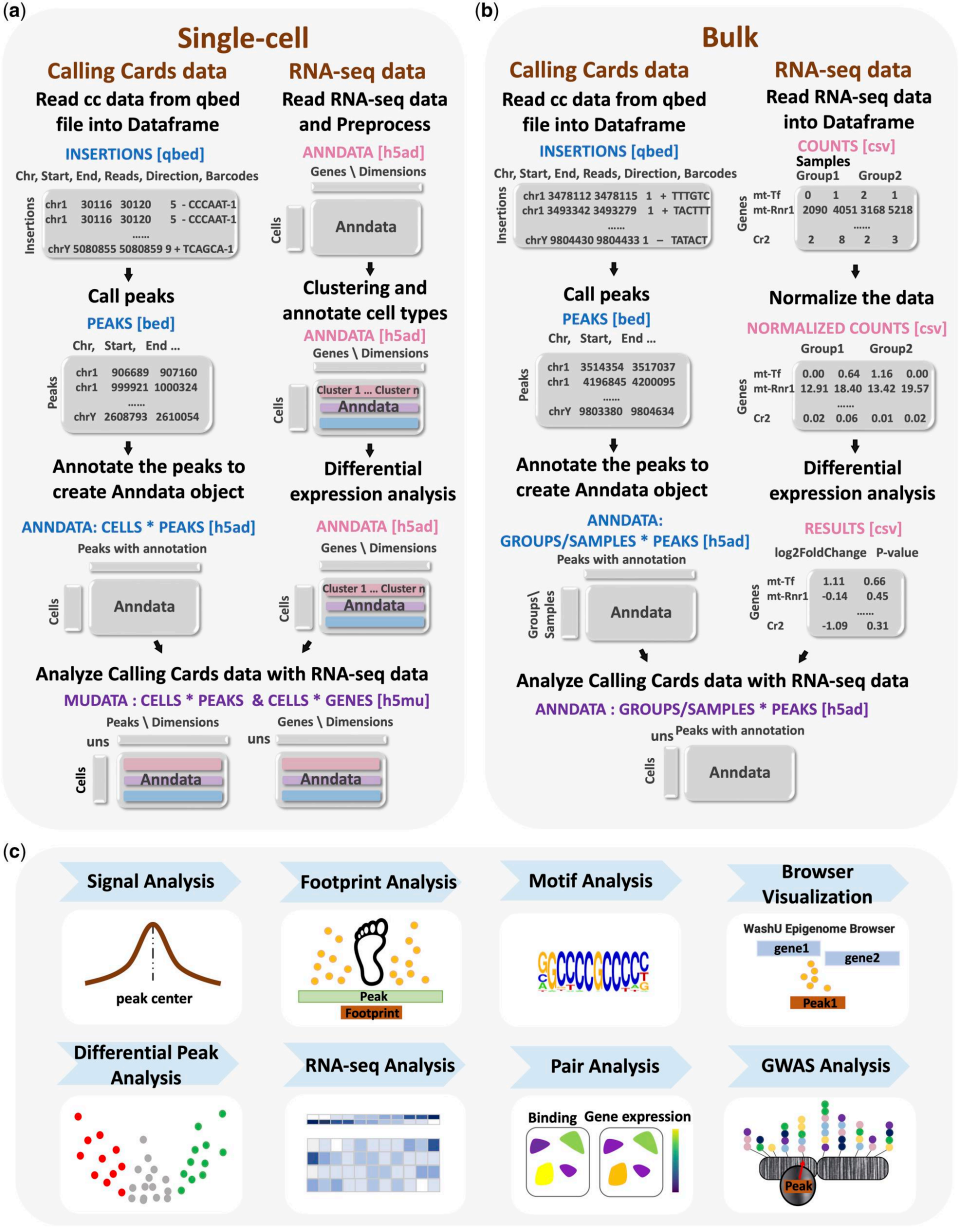
Overview of Pycallingcards. (**a**) Pycallingcards workflow for scCC data. Pycallingcards reads insertion data from a qbed file and then calls peaks (to create a bed file, left column). It then creates a cells-by-peaks Anndata object (h5ad file) Pycallingcards interfaces with Scanpy to complete preprocessing, clustering, and differential expression analysis of the RNA-seq data collected for each cell (right column). Pycallingcards then uses Mudata object to store the combined scCC and scRNA-seq data (h5mu file). (**b**) Data structure in Pycallingcards for bulk CC data. Pycallingcards reads insertion data from a qbed file and calls peaks, which generates a bed file. It later creates a groups/samples-by-peaks Anndata object (h5ad file) (b, left column). If bulk RNA-seq is provided, it uses normalized counts and results from differential gene analysis (b, right column). (**c**) Downstream Analysis. Pycallingcards provides functionality to compare called CC peaks with Chip-seq signal (when available), perform a footprint analysis to narrow down TF binding regions, find motifs, allow for visualization of the dataset through the WashU Epigenome Browser, perform differential peak analysis, pair CC data with RNA-seq data, and identify related SNPs by intersecting peaks with a GWAS database.

A schematic overview of typical Pycallingcards workflows for single cell and bulk CC is displayed in Fig. 1. Since the PBase transposase (PBase) naturally interacts with the Brd4 TF, by analyzing unfused PBase, one can map Brd4 binding across the genome. In order to map a specific TF of interest, transposons inserted by a TF-PBase fusion as well as unfused PBase are mapped separately; transpositions deposited by the TF-PBase are compared to those deposited by the unfused PBase, which is used as a background distribution. Regions that are enriched for TF-PBase insertions over background accurately reflect TF binding sites (Wang *et al.* 2012, Cammack *et al.* 2020, Moudgil *et al.* 2020). In scCC experiments, mRNA expression levels and the locations of transposon insertions are collected for each cell in a heterogeneous sample. Cells are first clustered by their mRNA expression profiles to identify the different cell types or subtypes present in the sample and then peaks of TF binding are called for each cluster. Peaks are then compared to identify genomic regions that are differentially bound between cell types. For bulk CC experiments, two or more conditions are typically analyzed with the primary goal being to call peaks in each condition to then identify differentially bound genomic regions.

For scCC data, Pycallingcards interacts with Scanpy (Wolf *et al.* 2018) and uses its functionality to analyze the scRNA-seq data and perform filtering, dimensionality reduction, and clustering of cells. Pycallingcards initially reads insertion data from a qbed file, where each line represents a single insertion and contains chromosome, insertion start site, insertion end site, number of reads, orientation of insertion, and the cell barcode associated with the insertion (detailed protocols to implement CCs, as well as to generate qbed files from raw sequencing reads are provided here; Moudgil and Mitra 2019, Moudgil *et al.* 2019). Normally, each dataset contains a large number of insertions. Pycallingcards peak calling methods aim to find putative TF binding sites by identifying genomic regions with higher-than-expected insertion densities. Three distinct methods for peak calling are available in Pycallingcards, each with several adjustable parameters; however, different methods and parameters typically yield fairly consistent results. We recommend plotting some genomic regions using the Pycallingcards Plotting submodule or by using the WashU Epigenome browser (Li *et al.* 2019, 2022) to tune the parameters and assess data quality. Users can then identify TF motifs enriched under a set of peaks using Homer (Heinz *et al.* 2010); this analysis can be used to evaluate the quality of the dataset, as one would expect to identify the motif of the TF being analyzed, but it can also be used to characterize the binding preferences of a TF for which no motif exists or to identify putative TF–TF interactions. Peak annotation is accomplished using bedtools (Quinlan and Hall 2010) and pybedtools (Dale *et al.* 2011), which are used to label each peak by finding the two closest genes. For scCC, a cells-by-peaks Anndata object for CC is generated, which contains information about insertion number, peak locations, and cell barcodes. The CC Anndata and RNAseq Anndata objects are then merged into one Mudata object in order to share data and conduct additional analyses (Bredikhin *et al.* 2022). Differential peak analysis among clusters can then be performed using several custom tools to identify peaks with significantly different numbers of CC insertions between clusters of cells. Pycallingcards also enables investigation of the relationship between TF binding and gene expression by integrating RNA-seq data with CC data to identify which peaks might control which genes. Finally, Pycallingcards provides tools for intersecting TF binding sites found by scCC with the GWAS Catalog (Buniello *et al.* 2019) to find TF binding sites with possible links to disease.

The workflow for bulk CC data analysis is identical to the scCC workflow for importing the data, peak calling, and annotation. At this step, a groups-by-peaks Anndata object is created according to the experimental condition (group) and then a differential peak analysis is typically performed for the different groups. This object can also track biological replicates within each group. If bulk RNA is also sequenced, it is often very informative to investigate the relationship between TF binding sites and the RNA expression levels of nearby genes across the various experimental conditions. Peaks identified through either scCC or bulk workflows can then be intersected with GWAS data.

Pycallingcards also has the ability to infer the exact binding sites of TFs, which can be especially useful for yeast data, where the TF often leaves a noticeable “footprint” so that insertions are deposited on either side of the binding site, but not at the site itself (Shively *et al.* 2019). For such data, we have included a function for footprint analysis that uses a Gaussian mixture model (Reynolds 2009) to identify the exact TF binding site.

### 2.2 Peak calling with Pycallingcards

Pycallingcards contains two novel methods for peak calling, CCcaller and MACCs. Both methods are inspired by aspects of the MACS2 package that is commonly used to analyze ChIP-Seq data (Zhang *et al.* 2008) but they are tailored specifically for use with CC data. These methods allow Pycallingcards users to call the peaks accurately, conveniently, and rapidly. Pycallingcards also contains an implementation of Blockify (Moudgil *et al.* 2020), which utilizes Bayesian Blocks (Scargle *et al.* 2013) to segment CC data.

Like MACS2 (Zhang *et al.* 2008), MACCs scans the genome using a specified window and step size and looks for regions with significantly more experimental insertions than background insertions. It merges consecutively enriched windows and then finds the center of this candidate peak. Next, MACCs determines whether the experimental insertions under the peak are significantly enriched over background by parameterizing a Poisson model using the number of background insertions observed under or adjacent to the peak. Since the PBase transposase inserts exclusively into TTAA tetranucleotides, MACCs accounts for the relative TTAA densities when comparing the number of transpositions across the different genomic regions. Candidate peaks that pass a *P*-value threshold are then considered bona fide CC peaks. There is also a background-free version of this algorithm that computes significance by comparing the number of transpositions under the candidate peak to the number of insertions in the neighboring genome defined by lambda window size (further details are included in the Supplementary Information).

CCcaller differs from MACCs in that it uses a greedy algorithm (Edmonds 1971) to identify candidate peaks. CCcaller starts from first insertion on each chromosome and extends the candidate peak if the next nearest insertion is within a user-specified number of base pairs. Candidate peaks are then promoted to CC peaks exactly as described for the MACCs method. The CCcaller method is computationally efficient because every insertion is only considered once. It also covers as many insertions as possible because it tries to include every insertion as part of a peak.

To compare the performance of different methods, we compared five different methods: CCcaller, MACCs, Blockify in Pycallingcards (Blockify), the original Blockify (Blockify_original), and ccf_tools. For each method tested, we used the default parameters. We downloaded CC data from GEO (see Supplementary Table S1). We used HyPBase Brd4 bulk CC data in HCT116 to examine peak calling performance for data without background and SP1 scCC data in HCT116 to test peak calling performance for CC data with background.

**Figure 2.**
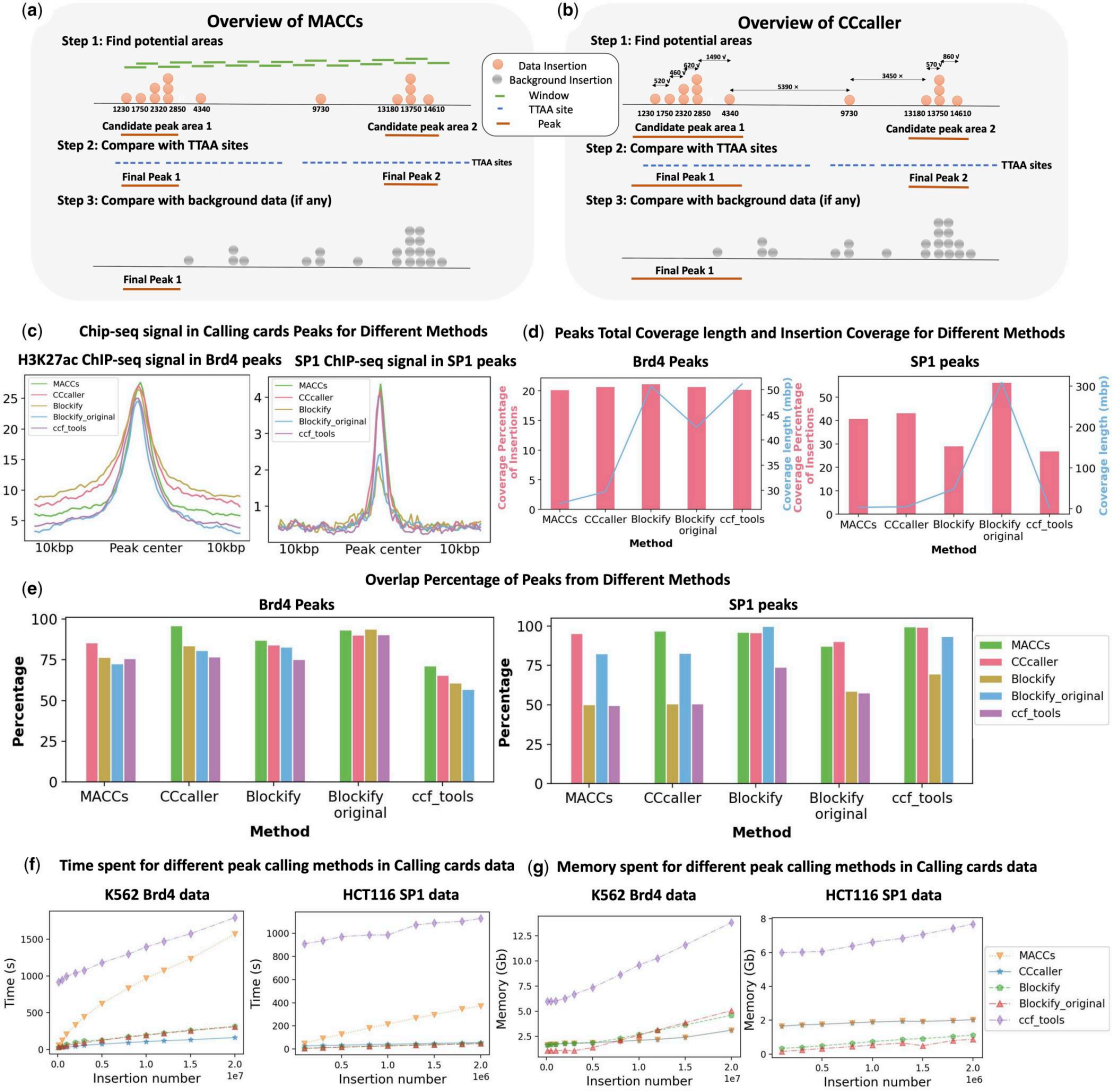
Peak calling methods for Calling cards data. (**a**) Overview of MACCs. (**b**) Overview of CCcaller. (**c**) Chip-seq signal in calling cards peaks for the different peak calling methods in Pycallingcards (left: peaks calling without background, HCT116 Brd4 data; right peaks calling with background, HCT116 SP1 data). (**d**) The percentage of insertions contained under all called CC peaks is plotted for different methods (pink bars). On the same plot, the cumulative peak length of all called peaks is plotted (blue bars, left: peaks calling without background, K562 Brd4 data; right peaks calling with background, HCT116 SP1 data). (**e**) Percentage of peaks that overlap between the different peak calling methods. [left: K562 Brd4 data (no background peak calling); right: HCT116 SP1 data (peaks calling with background)]. (**f**) Computational time required for each of the different peak calling methods in Pycallingcards [left: K562 Brd4 data (no background peak calling); right: HCT116 SP1 data (peaks calling with background)]. (**g**) Memory required for different peak calling methods in Calling cards data (left: peaks calling without background, K562 Brd4 data; right peaks calling with background, HCT116 SP1 data).

To benchmark the different peak callers, we plotted the average Chip-seq signal (ENCODE Project Consortium *et al.* 2012, Zhang *et al.* 2020) under the different sets of called peaks (see Fig. 2c). We first compared Brd4 CC peaks with H3K27ac signal measured by ChIP-seq. Since Brd4 binds at H3K27ac histones, we expect strong enrichment of ChIP-seq signal under the called peaks. We saw strong enrichment of H3K27ac signal under peaks called by all five methods, with only minor differences in performance. However, when SP1 scCC data were analyzed and compared to a previously published SP1 ChIP-seq dataset (Zhang *et al.* 2020), MACCs, CCcaller, and ccf_tools outperformed Blockify (see Fig. 2c right panel). This can be partly explained by the fact that Blockify tends to call much wider peaks than the other methods, despite the fact that the total number of insertions assigned to a peak is similar across methods (Fig. 2d). Blockify (as well as Blockify_original) often calls extremely wide peaks for CC data with low numbers of insertions (e.g. single-cell data, see Supplementary Fig. S2), so in such instances, we recommend using MACCs or CCcaller. To better understand the concordance between the different peak calling methods, we plotted the percentage of peaks called by each method that overlap with a peak called by a different method (Fig. 2e). CCcaller and MACCs call very similar sets of peaks, while ccf_tools, Blockify, and Blockify_original are more discordant. Since CCcaller and MACCs also showed the highest enrichment with ChIP-seq signal (Fig. 2c), our analysis suggests one of these should be the peak calling method of choice (see also Supplementary Table S9). We also called motifs for peaks from SP1 scCC data and SP1 ranked at the top for every method. To better quantify the result, we designed a simulation with authentic peaks (directed insertions) and “background” peaks (undirected insertions into TTAAs), allowing us to systematically benchmark the various peak calling methods against this standard in the Supplementary Information.

We next benchmarked the five peak calling algorithms on their computational performance. To determine how their runtimes scale with dataset size, we performed two separate experiments. First, to benchmark background-free peak calling, we mapped PBase insertions (directed by the Brd4 protein) in the K562 cell line (see Fig. 2c and d), using >20 million transposition events, the largest dataset of transposon insertions produced to date. Then, to benchmark peak calling with a background distribution, we used previously published SP1 and Brd4 CC data collected from HCT116 cells (Moudgil *et al.* 2020). We downsampled the data, called peaks, and plotted the runtime versus total insertion number for each method. CCcaller runs significantly faster than the other methods, running 7–10× faster than MACCs and ccf_tools and 2- to 3-fold faster than Blockify when processing datasets with more than five million insertions (Fig. 2f), while requiring very little memory (Fig. 2g). Taken together, our results suggest that CCcaller and MACCs are the most accurate peak callers and that CCcaller is the most computationally efficient algorithm and can be used to call peaks for large CC datasets in <5 min on a personal computer.

### 2.3 Differential peak analysis

It is often important to identify regions of the genome that are differentially bound by a TF in two different biological conditions. Pycallingcards provides functionality to compare TF binding sites across two samples (or cell types, for scCC) and find peaks where there is significantly more binding in one sample than the other. This can be challenging as the samples may have slightly shifted peaks centers at a given genomic region, leading to false positive differential peak calls. Pycallingcards employs two different strategies to deal with this problem. In the first strategy, Pycallingcards combines the insertions from the samples and calls peaks on the joint dataset. Then, a Fisher’s exact test or binomial test is utilized to determine if the number of insertions under each peak is significantly different from others. In the second strategy, peaks are called separately in each sample and then overlapping peaks are combined between samples and then significance tests are performed on the combined peaks as above. The details and workflow of this analysis can be found in the Supplementary Information.

### 2.4 Integrating CCs with RNA-seq

In scCC experiments, scRNA-seq data are always collected in parallel with CC insertion data. For bulk CC experiments, it is often useful to collect RNA-seq data either from the same biological samples under different experimental conditions or from cells with the TF of interested knocked out compared to wild-type control cells. When such data are available, it enables the integration of CC data with the corresponding RNA-seq data to identify which TF binding events affect the transcription of nearby genes and to quantify the magnitudes and directions of the effects.

For scCC experiments, Scanpy is utilized for the normalization, filtration, dimensionality reduction, clustering, and identification of differentially expressed genes from scRNA-seq data (reference Fig. 1). Next, regions of the genome that are differentially bound are identified (using a Fisher’s exact test or binomial test), allowing for the direct comparison of changes in TF binding with the gene expression changes of nearby genes across different cell types. For bulk CC experiments, differential peak analysis is performed to identify changes in TF binding across the different experimental groups. Then either the DEseq2 (Love *et al.* 2014) or PyDESeq2 (Muzellec *et al.* 2022) is used to find differential genes in bulk RNA-seq data. Next, for each experimental group, correlations between TF binding and gene expression changes can be readily identified. Such correlations can be used to infer the gene targets, and thus biological roles, of the analyzed TF.

The CC-peak/DE gene pairs can be used to generate specific hypotheses. For example, often TF binding correlates with the activation of a nearby gene that plays an essential role in disease or is expressed in a specific cell type. To quickly reveal such connections, we can search the GWAS Catalog (Buniello *et al.* 2019) to find all SNPs within CC peaks to generate hypotheses about the mechanism by which genetic variation might play a role in disease. For the mouse genome, the Liftover (Hinrichs *et al.* 2006) tool (with some modifications) is used to map from the mouse genome (mm10) to the human genome (hg38) to determine if any trait-associated SNPs exist in the orthologous human genomic region.

### 2.5 Additional functionality in Pycallingcards

Pycallingcards has a variety of other useful tools for the analysis of CC data. For example, it can compare CC peaks with signals from different -omics datasets (e.g. Chip-seq, RNA-seq), to validate peak calls and to extract additional biological inferences from CC experiments. It can also calculate and plot the signal around peaks and make heatmaps. Pycallingcards can also provide a genome-wide view of CC peaks by plotting peak density along whole chromosomes to give an overview of the dataset. It is also designed to easily connect with WashU Epigenome Browser to produce publication quality figures and visualize CC data against a backdrop of different genomic features or hundreds of orthogonal datasets. Additionally, for yeast CC data, footprint analyses can be performed to find more accurate TF binding sites. We highlight the utility of the tools available in Pycallingcards through a set of vignettes using real data below.

### 2.6 Analysis of single-cell CCs mouse cortex data

**Figure 3.**
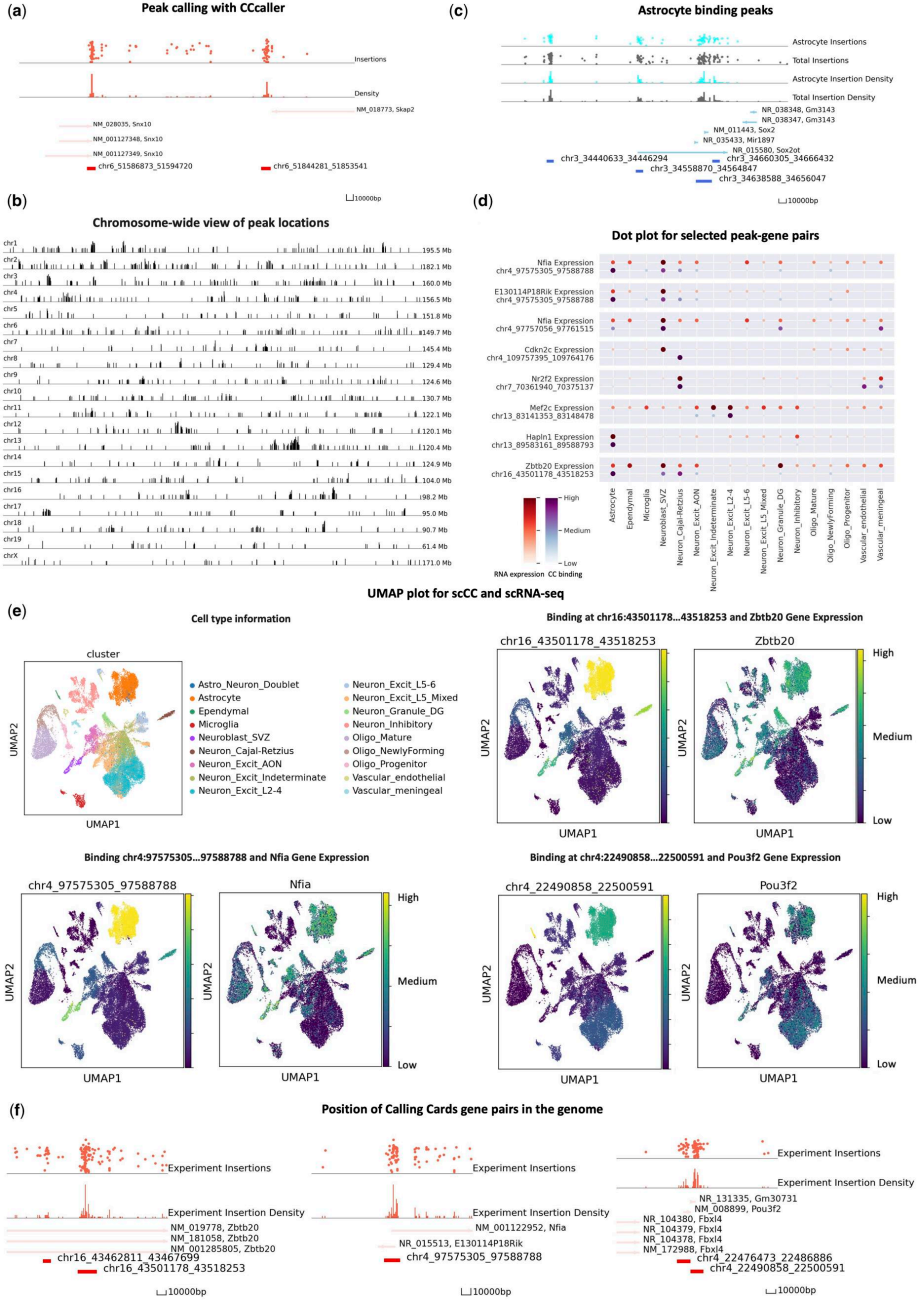
Application of Pycallingcards to single-cell mouse cortex data. (**a**) Peak calling with CCcaller. The top two tracks of the plot are the insertion data. They show the exact positions of the insertions with the *y*-axis as log reads number. The middle two tracks display insertion density. The bottom track shows nearby genes and the locations of called peaks. (**b**) The distribution of peaks throughout the chromosomes. Each peak is represented by a line whose height is proportional to the log of the insertion number. (**c**) Selected peaks significantly bound in astrocytes, compared to total data (gray). (**d**) Dot plot of selected peak–gene pairs. (**e**) UMAP plot for scRNA-seq data of cluster and peak–gene pairs. The UMAP plot for CC data in € is colored according to TF binding across the whole cluster. (**f**) Position of and peak–gene pairs in the genome.

To demonstrate the core functions and procedures of Pycallingcards for single-cell data, we reanalyzed the Brd4 scCC dataset in the mouse cortex (Moudgil *et al.* 2020) (see Fig. 3). In this dataset, there are 111 382 insertions and 35 950 cells in total. For each cell, mRNA expression levels and CC insertions are recovered, providing insights into the gene regulatory networks that guide cell fate specificity and homeostasis.

To analyze this dataset, we first used Scanpy to filter cells and genes, reduced the data dimensionality, and clustered cells to identify their cell types. This analysis identified six cell types and 18 different subtypes. Clusters were assigned to cell-types by referring to the original paper (Moudgil *et al.* 2020) and these assignments were stored in an Anndata object. Next, CCcaller was used to call peaks (see Fig. 3a). Each Brd4 binding site is captured as a peak, and a total of 902 peaks were called. The locations of these peaks are visualized across the genome in Fig. 3b, using the draw_area function. Each peak is then annotated with the two nearest genes using the annotation function.

A cells-by-peaks Anndata object was then created from the mapped insertions, called peaks, and cell barcode data. The scRNA-seq object (created using Scanpy) and the scCC Anndata object were then combined with each other to connect TF binding and mRNA expression information for every single cell. Using the rank_peak_groups function in Pycallingcards, binomial tests were then used to identify cell type-specific TF binding, and *t*-tests were used to discover genes with cell type-specific expression (see Fig. 3c).

We next used binomial test to look for differentially bound peaks across cell types whose neighboring genes displayed gene expression changes across the same cell type (log foldchange g ⩾ 3 and adjusted *P*-value  ⩽ .05). This analysis identified many peaks whose binding correlated with the expression of nearby genes in a cell type specific manner, suggesting these regions represent Brd4-bound enhancers that regulate the associate genes (see Supplementary Notes and Supplementary Table S2). For example, the cell types with the strongest Brd4 binding at peak chr13:83141353…83148478 also have high expression of the nearby gene Mef2c (e.g. in excitatory neurons), and the strong Brd4 binding at chr4:97575305…97588788 in astrocytes and subventricular zone derived neuroblasts is correlated with higher expression levels of the nearby gene E130114P18Rik in these cell types (see Fig. 3d).

The ability of Pycallingcards to visualize and integrate different types of data enables rapid hypothesis generation. For example, in reanalyzing the mouse cortex data with Pycallingcards, we identified a putative regulatory circuit controlling astrocytogenesis. The TFs Nfia and Zbtb20 are known to bind cooperatively to bind at multiple loci to promote astrocytogenesis. In particular, these TFs are known to directly suppress the expression of Pou3f2 (Medeiros de Araújo *et al.* 2021), which encodes a protein required for upper-layer neuron specification. However, the cis enhancers controlling the expression these genes are not known. We found a Brd4 binding peak adjacent to Nfia on chromosome 4 (peak chr4:97575305…97588788) that is correlated with its expression (see Fig. 3e). Similarly, a binding peak adjacent to Zbtb20 at chr16:43501178…43518253 appears to regulate Zbtb20 expression in astrocytes. Finally, there is a binding site at chr4:22490858…22500591 whose Brd4 binding correlates with Pou3f2 expression (high in excitatory neurons, lower in astrocytes), leading to the hypothesis that Nfia and Zbtb20 block the activity of the Pou3f2 enhancer in astrocytes, but not in excitatory neurons locus and this differential binding contributes to fate specification. Further GWAS analyses are included in the Supplementary Information.

### 2.7 Analysis of bulk GBM CCs data

Pycallingcards also has multiple functions to facilitate the analysis of bulk data. We illustrate this by analyzing two wild-type (Brd4-directed) CC datasets obtained using a model of GBM derived from murine neocortical postnatal Day 1 astrocytes engineered with a combined loss of function for neurofibromin (NF1) and p53. This model is useful for studying sex differences, as male- and female-derived cells from littermates are isogenic except at the sex chromosomes (Kfoury *et al.* 2021), yet have quite a differential cellular phenotype in tumor assays.

**Figure 4.**
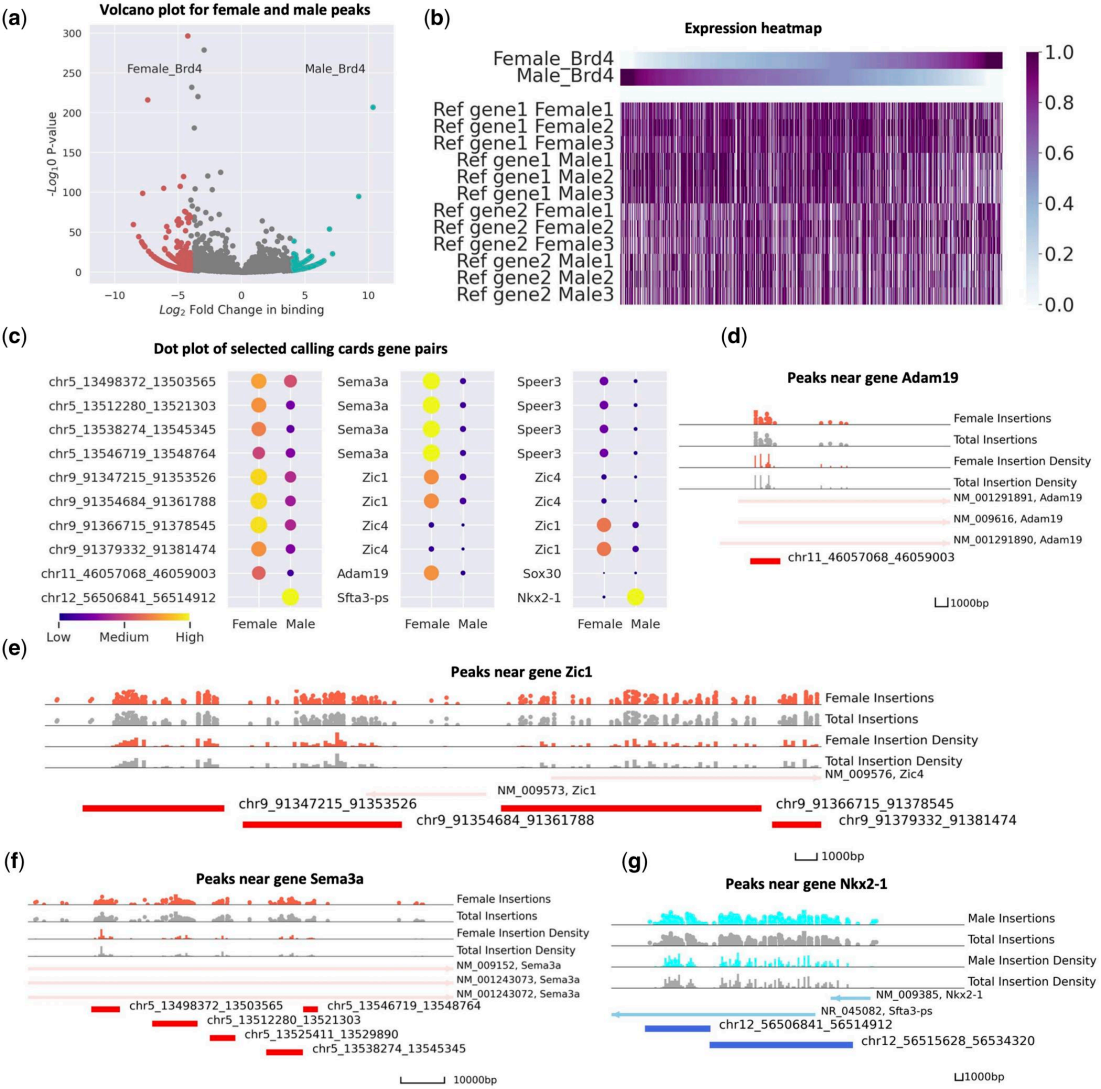
Application of Pycallingcards to bulk glioblastoma CC data. (**a**) Volcano plot for female and male peaks. The dots on the left-hand side indicate peaks with more Brd4 binding in females and the dots on the right hand display more Brd4 binding in males. The *x*-axis is the log fold change and the *y*-axis is the log *P*-value. (**b**) Heatmap for calling cards and RNA-seq expression. The first two lines plot the relative Brd4 binding in males and females at differentially bound peaks. The following lines display the relative gene expression of the nearest (first six rows) and next-nearest (second six rows) genes. (**c**) Dot plot of selected calling cards gene pairs. (**d–f**) Peaks near gene Sema3a, Adam19, and Zic1. Red symbols and density plots represent Brd4 insertions and insertion density in females, whereas gray symbols and plots represent Brd4 binding in males. (**g**) Peaks near gene Nkx2-1. Blue symbols and density plots represent Brd4 insertions and insertion density in males, whereas gray symbols and plots represent Brd4 binding in females.

We used the CCcaller with a maxbetween value as 1100 to call peaks on the joint dataset and then used the annotation function to annotate the peaks with the two nearest genes, resulting in a groups-by-peaks Anndata object. We identified regions of the genome that were differentially bound by Brd4 by performing Fisher’s exact tests and used the volcano_plot function to visualize the differentially bound peaks (Fig. 4a). Interestingly, we found that across the genome females were more likely to have a differentially bound peaks with increased Brd4 binding relative to male samples. We next compared the CC data to bulk RNA-seq data for males and females, which were collected in triplicate. Using the pair_peak_gene function, we compared the expression of genes near differentially bound peaks in Fig. 4b. We found that if a peak displays more Brd4 binding in one sex, then the gene expression of the nearby genes are more differentially expressed in that sex (*P*-value <.001), and this effect is stronger for the gene closest to the enhancer (first two rows) than for the second closest gene (third and fourth rows). Next, we used DEseq2 to find genes that are differentially expressed in males and females to find genes that have significant differences in both Brd4 binding and gene expression. The top genes are shown in Fig. 4c. We then plotted the relative Brd4 binding in males and females at each of these loci (Fig. 4d–g). Brd4 directed CC insertions into female cells are colored red and insertions into male cells are colored blue. We found that Sema3a, Adam19, and Zic1 all have significantly more Brd4 binding (and RNA expression levels) in female cells than in male cells (see Fig. 4d–f). All of these genes are associated with poor outcomes in GBM (Salero *et al.* 2001, Vadasz *et al.* 2018, Angelucci *et al.* 2019), and suggest that these genes may represent sex-specific therapeutic targets. In contrast, we observe more Brd4 binding near Nkx2-1 (also known as TTF1) and higher expression of this gene in male GBM cells, Nkx2-1 has been reported to promote metastasis and its expression is associated with a molecular subtype of IDH-wildtype GBM (Prok and Prayson 2006, Wildeboer *et al.* 2006, Han *et al.* 2021, Suwala *et al.* 2021), which has a poor prognosis. Thus, Brd4 binding at Nkx2-1 may contribute to the disproportionately poor outcomes observed in male patients relative to female patients (see Fig. 4g).

We can also examine the sequence of the peaks to try to predict from their motifs the TFs driving the sex differences in Brd4 binding (and thus gene expression). Fisher exact test is used for differential peaks analysis; female and male samples are selected and motifs are called separately to identify significantly bound motifs in each sex (see sub). Four TF families, IRF (ISRE, IRF2, IRF3, IRF8, IRF1, IRF4, etc.), ETS (GABPA, ETS1, Elf4, ETV1, Elk1, etc.), TEA (TEAD3, TEAD, etc.), MADS (Mef2c, Mef2a, CArG), are found in female but not in male CC data. In terms of male samples, the HMG (Sox3, Sox10, Sox4, Sox2) family shows an extreme difference in motifs from our data (see Supplementary Notes and Supplementary Tables S4 and S5).

## 3 Discussion

Pycallingcards is the first bioinformatics tool to provide a comprehensive, efficient, and easy-to-use set of functions for the analysis and visualization of CC data. It provides tools to integrate CC data with RNA-seq data in order to link genomic peaks to the genes they regulate. The application of Pycallingcards to CC datasets allows users to rapidly generate new hypotheses about the effects of TF binding on gene expression. Thus, Pycallingcards facilitates the analysis and interpretation of CC data.

Through the exploration of the mouse cortex data via Pycallingcards, we illustrated new hypotheses about genomic regions specifying specific cell types and subtypes. Further study can be done on the Brd4 regulation of Zbtb20 in mammalian neocortical progenitors and postmitotic cells to determine if this binding influences cell fate specification. For example, the regulatory regions that we identified could be targeted using enhancer-targeting CRISPR-epigenetic editing to determine their roles in astrocytogenesis. Using Calling Cards, Kfoury *et al.* (2021) were able to identify sex differential BRD4-bound enhancer usage that promotes epithelial-to-mesenchymal transition and cancer stem cell proliferation in males. By integrating bulk RNA-seq data with Pycallingcards, sex-specific BRD4 Calling Cards peak regions were associated with the target genes. Additionally, differential motifs were identified. This data can be used for further investigation into the therapeutic effect of BET inhibitors in different cancer models. Altogether, this demonstrates the utility of Pycallingcards for the facile analysis of CC data. Our reanalysis of published data generated several novel hypotheses, such as the role of Nfia and Zbtb20 binding at the Pou3f2 enhancer in astrocytogenesis, novel binding of Tye7p upon gcr2 knockout, and novel TF families that may be involved in sex difference in GBM that were not identified in the original publications (see Supplementary Notes and Supplementary Table S6). Furthermore, these analyses could be completed in one integrated environment, greatly simplifying workflow.

There are two limitations to the current work. First, as this is meant to be tutorial and description of this new package, we did not conduct extensive biological validation of predictions made here. Those studies will be better suited to other formats. Second, we presented here only nonparametric methods for differential peak analysis (binomial testing). Future studies will need to investigate the distributions of CC data and the assumptions of parametric approaches to determine if these may further improve the sensitivity and specificity of detecting differential insertion sites. Nonetheless, given the flexibility of the python framework, future methods should be readily incorporated into the package, which will be maintained at the Github (https://github.com/The-Mitra-Lab/pycallingcards).

Finally, because Pycallingcards integrates with Scanpy and the Python data science environment, there is now the potential to leverage the multitude of python-based deep learning APIs to create novel methods for the analysis of multi-omics biological data. Additionally, as more CC data are produced, we will better understand the distributions of CC data and the variance among samples and cells, allowing for better null models and more accurate statistical analyses for differential peak calling. In the future, CC might also be able to combine with other single cell genomic measurements, such as ATAC-seq, spatial RNA-seq, and single cell HiC. Pycallingcards provides a foundation upon which future analytical packages can build for all of these technologies and enables the efficient downstream analysis of all types of CC data.

## 4 Material and methods

Detailed methods are provided in the Supplementary Information. Tutorials and example jupyter notebooks are available at https://github.com/The-Mitra-Lab/pycallingcards.

## Supplementary Material

btae070_Supplementary_Data

## Data Availability

Raw and processed data generated in this study have been deposited to NCBI Gene Expression Omnibus with the accession number GEO: GSE248420. The calling cards data are publically available datasets analyzed in this study and can be downloaded from the GEO with accession codes GSE148448 and GSE156821. The Chip-seq data are publicly available datasets analyzed in this study and can be downloaded from ENCODE [ENCFF997CJQ, ENCFF587ZMX]. Access to the genome annotation used: mm10 [ftp://hgdownload.soe.ucsc.edu/goldenPath/mm10/] and hg38 [ftp://hgdownload.soe.ucsc.edu/goldenPath/hg38/].

## Code availability

Pycallingcards is available through Github (https://github.com/The-Mitra-Lab/pycallingcards) and the documentation is available at https://pycallingcards.readthedocs.io/en/latest/.
