# Supplementary material for: Pycallingcards: an integrated environment for visualizing, analyzing, and interpreting Calling Cards data
Source: Bioinformatics. 2024 Feb 7;40(2):btae070. doi: 10.1093/bioinformatics/btae070 (PMC10881108; doi:10.1093/bioinformatics/btae070)
Supplement: btae070_Supplementary_Data [file btae070_supplementary_data.pdf]

Methods

Package design

We aimed to create a user-friendly and extensible platform for the reading, prepossessing, analyzing, and visualization of CC data. Pycallingcards takes as input a text file containing mapped transposon insertions (in qbed format (Moudgil et al., 2021)) and calls peaks, annotates them, finds TF motifs over-represented near binding sites, performs differential peak analysis, enables the comparison of TF binding and RNA expression, allows for the intersection of disease-associated loci found in GWAS with peak calls, and lets the user visualize each step in this process. Pycallingcards is organized into five submodules: the Datasets submodule, which contains several exemplar CC datasets; the Reading submodule, which allows for the reading, writing, and merging of different file formats (e.g. qbed, barcode files, RNA-seq data files, etc); the Preprocessing submodule which contains functions to clean qbed files, call TF binding peaks, and annotate them; the Tools submodule, which contains tools for the downstream analysis of CC peaks such as differential peak calling, integration with RNA-seq data, TF footprint identification, comparison with ChIP-seq data, and integration with GWAS datasets; and the Plotting submodule, which enables visualization of calling card insertions and integration with the WashU epigenome browser to produce publication-ready figures. This submodule also provides methods for the visualization of the different downstream analyses performed by the Tools submodules.

Pycallingcards builds on the Anndata (Wolf, Angerer, & Theis, 2018) and Mudata (Bredikhin, Kats, & Stegle, 2022) objects that are widely used for the analysis of single cell data. For CC data, Pycallingcards creates a cells/samples-by-peaks matrix which allows the user to interact with different multi-omics analysis tools. The Anndata object includes a table for peaks that stores peak annotation information, a table for cells or samples that indicate types, and a matrix that stores binding information. Other results (e.g. differential peaks calls) can also be stored in this object. For integrated multi-omic analysis, Pycallingcards combines the two Anndata objects that hold CC and RNA-seq data into a single Mudata object. This facilitates the discovery of TF binding events that are correlated with gene expression and allows the user to correlate binding and gene expression with SNP and GWAS information, which is typically contained in pandas data frames and saved to the Anndata or Mudata objects.

Peak calling

Pycallingcards supports three different peak calling methods, CCcaller, MACCs, and an optimized version of Blockify. These methods identify regions in the genome for which there are significantly more TF-directed insertions than background (PBase) insertions. The peak calling workflows for CCcaller and MACCs are shown in Fig 2a and 2b. The Blockify algorithm is described in detail elsewhere(Moudgil et al., 2020). CCcaller and MACCs each have three steps and only differ in the first one. In the first step, CCcaller identifies candidate TF binding sites by considering a candidate peak at the first insertion on each chromosome and extending the width of the candidate peak to include other insertions until there are no insertions nearby. In contrast, MACCs, which is modeled after the popular ChIP-Seq analysis programs MACS and MACS2, uses a sliding window to identify candidate peaks, starting at the first TF-directed insertion on a chromosome. The second step in both algorithms is testing whether there are significantly more TF-directed insertions than background insertions in this potential area by applying the Poisson test using a lambda value parameterized from insertion density in the surrounding area (optionally with user-specified pseudocounts). The definition of p-value here is similar to MACS2 (Y. Zhang et al., 2008). More precisely, we estimate the number of insertion numbers In_i and the number of TTAA sites T_i in the surrounding area whose length is defined by lam_win_size. We then calculate the number of insertions in_i and the number of TTAA sites t_i in this potential area. The expected number of insertions λ_i in this potential area is $\frac{In_i * t_i}{T_i} + psuedocounts$, Poisson test is tested on $in_i + pseudocounts$ to find out if it is a peak or not. For MACCs, if significance is achieved and the window is extended by a user-specified step size, the test is repeated until the candidate peak can no longer be extended. Finally, the algorithms then compare the experimental data to the density of background insertions around the peak, while adjusting for TTAA density. To determine significance, a Poisson test similar to that described above is performed to test whether the CC insertions under the peak are at a significantly higher density than expected from the background data. MACCs then centers the final peak coordinates on the distribution of insertions. For experiments where no background data is available, both CCcaller and MACCs support background-free peak calling. The algorithms scan through the genome in the same manner as described above, but the null models are parameterized by the average insertion density in a large window centered on the candidate peak (after adjusting for relative TTAA densities in the two windows). In this manner, regions with high local densities of insertions are identified. When calling peaks, we treat insertions as

independent events and only record the start point of insertions. This allows us to reduce the time complexity of the algorithm to $O(n)$ if the qbed data is sorted, substantially reducing the number of computing resources needed.

Footprint analysis

Pycallingcards provides functionality to compute TF footprints, which is useful for the analysis of yeast CC data, which, unlike mammalian CC data, has single nucleotide resolution. By analyzing the pattern of insertions in the yeast genome, one can often precisely estimate the binding site of the TF. This is accomplished using a Gaussian mixture model. This model assumes that distributions of insertions to the left and right of a binding site follow two independently parameterized Gaussian distributions: r_i represents the data from the right distribution and l_i represents the data from the left distribution where $r_i \backsim N(r, \mu_r, \sigma_r) = \frac{1}{\sigma_r\sqrt{2\pi}}e^{-\frac{(r-\mu_r)^2}{\sigma_r^2}}$ and $l_i \backsim N(l, \mu_l, \sigma_l) = \frac{1}{\sigma_l\sqrt{2\pi}}e^{-\frac{(l-\mu_l)^2}{\sigma_l^2}}$ where $\mu_r \neq \mu_l$. Combining the two Gaussian distributions, the equation becomes: $p(x \mid \mu_r, \mu_l, \sigma_r, \sigma_l, \pi_l, \pi_r) = \pi_r N(x, \mu_r, \sigma_r) + \pi_l N(x, \mu_l, \sigma_l)$.

The parameters of the model are then estimated by expectation-maximization (EM) (McLachlan & Krishnan, 2007). EM is a two-step iterative algorithm that alternates between performing an expectation step, where expectations for each data point are computed based on current parameter estimates by $p_{ic} = \frac{\pi_c N(x_i|\mu_c,\sigma_c)}{\pi_r N(x_r|\mu_r,\sigma_r)+\pi_l N(x_l|\mu_l,\sigma_l)}, c \in r, l, i \in 1, ..., n, n$ is the total number of data in the peak. In the maximization step, we update $\mu_r, \mu_l, \mu_r, \sigma_l, \sigma_l$ based on the maximum likelihood estimate. After the algorithm converges, μ_r, μ_l are found and we report the area between the mean values of each distribution as the true binding site.

Signal calculation

In order to compare the signal of CC data with other reference sequences, eg Chip-seq, Cut & Run data, Pycallingcards, calculate a running average of the signal around the peak. The default settings use a 20000 bp window centered on the peak with a bin size of 100.

Motif analysis

HOMER (Heinz et al., 2010) is utilized for motif analysis in Pycallingcards. It incorporates a unique motif discovery technique aimed at genomics applications including regulatory element analysis. It is a differential motif discovery method, which attempts to uncover regulatory components that are disproportionately abundant in one set of sequences compared to the other. Motif enrichment is determined by combining zero or one occurrence per sequence (ZOOPS) score, with hypergeometric enrichment calculations. HOMER gives a rank of all of the motifs and finds the most significant binding motifs.

Annotation

Pycallingcards uses Bedtools (Quinlan & Hall, 2010)to find the two closest genes to each called peak. The closest function in Bedtools searches for overlapping areas in the peaks and reference data. In the event that no feature in reference gene overlaps the peaks, closest will report the nearest (that is, least genomic distance from the start or end of peaks) gene (Quinlan & Hall, 2010). After the peaks are annotated, the information is stored in an Anndata object, for cells/groups-by-peaks X_{n*p}, n represents the number of cells/groups and p indicates the number of peaks, which is produced by CC data and peak data.

Differential peak analysis

The goal of differential peak analysis is to compare TF binding sites across two samples and find peaks where there is significantly more binding in one sample than in the other. This can be challenging as two samples may have slightly shifted peak centers at a given genomic region, leading to false positive differential peak calls. Pycallingcards employs two different strategies to address this problem. In the first strategy, the two samples to be compared are combined and peaks are called on the joint dataset. Next, a Fisher's exact test or binomial test is utilized to determine if the number of insertions under each peak from one sample is significantly different the number of insertions under the peak from the other sample. In the second strategy, peaks are called separately for each sample and overlapping peaks

are combined between samples and then significance tests are performed on the combined peaks exactly as before. To better illustrate this process, Fig.3 shows the workflow of the analysis.

When analyzing scCC data, one is generally interested in identifying cell-type specific binding. Thus, Pycallingcards computes the probability that the TF binding in one cell type is significantly higher than in the average of all other cell types; as such, one-sided tests are used. For example, when using Fisher's exact test, for each peak and each cell type we have the variables c_A (number of cells in the peak of the specific cell type), c_B (number of cells in the peak of the rest of cell types), n_A (number of cells in the other peaks of the cell types), n_B (number of cells in the other peaks of the rest cell types). The probability of each circumstance is $p = \frac{(c_A+c_B)!(n_A+n_B)!(c_A+n_A)!(c_B+n_B)!}{c_A!c_B!n_A!n_B!(c_A+c_B+n_A+n_B)!}$. The null hypothesis is that the expected number of TF-directed insertions in the cell type of interest is the same as the expected number of TF-directed insertions in the other types. In the binomial test, for each peak and each type, we have i_A (number of insertions in the peak of type), i_B (number of insertions in the peak of the rest cell types), t_A (number of cells in all the peaks of the types), and t_B (number of cells in all the peaks of the rest cell types). Under the null hypothesis, the probability of insertion number in the peak of type $p(k) = \binom{k}{i_A+i_B}(\frac{t_A}{t_A+t_B})^k(1-\frac{t_A}{t_A+t_B})^{i_A+i_B-k}$. We assume that the insertions capture the same ratio of cell numbers.

When analyzing bulk CC data, one is usually most interested in finding peaks that are differentially bound between two samples that represent different treatments, cell types, or time points. As a result, two-sided tests are used for differential peak calling for bulk CC data. For example, when using the Fisher's exact test, for each peak and each group we have the variables i_A (number of insertions in the peak of the group), i_B (number of insertions in the peak of the other group), n'_A (number of insertions in the other peaks of the group), and n'_B (number of insertions in the other peaks of the other group). The probability of each circumstance is $p = \frac{(i_A+i_B)!(n'_A+n'_B)!(i_A+n'_A)!(i_B+n'_B)!}{i_A!i_B!n'_A!n'_B!(i_A+i_B+n'_A+n'_B)!}$. Here, the null hypothesis is that the expected number of TF-directed insertions under the peak in one sample is the same as the expected number of TF-directed insertions in the other types. In the binomial test, for each peak and each group, t_A (number of insertions in all the peaks of the group), and t_B (number of insertions in all the peaks of the rest group). Under the null hypothesis, the probability of insertions number in the peak of group $p(k) = \binom{k}{i_A+i_B}(\frac{t_A}{t_A+t_B})^k(1-\frac{t_A}{t_A+t_B})^{i_A+i_B-k}$. We assume that the insertions should capture the same ratio of total insertion numbers.

After all the tests are completed for each peak for one group/cell type, Benjamini-Hochberg correction (Thissen, Steinberg, & Kuang, 2002) is followed to calculate the adjusted p-value.

Pair analysis

Pycallingcards can group peaks that are bound in a cell-type specific manner to nearby genes that are expressed in a cell type specific manner, which then become peak-gene pairs. This functionality is useful for exploring the transcriptional consequences of TF binding.

For scCC data, peaks for which binding is significantly enriched in one cell types are paired with genes significantly expressed in the same cell type using the pair_peak_gene function, which takes as input a specific cell type (or groups of cell types), (adjusted) p-value cutoffs, and log fold change cutoffs for both the differential gene analysis and differential peak analysis. If the peak and the annotated gene are significant, they are annotated as matched peak-gene pairs.

GWAS analysis

Pycallingcards also provides functionality to determine whether significantly bound peaks are near SNPs associated to disease in a GWAS study. In GWAS analysis, all SNP data are downloaded from the GWAS Catalog (`https://www.ebi.ac.uk/gwas/docs/file-downloads`) and are stored by cell type/group. First, a peak by SNP sparse matrix D_{p*s} is created, where p is the number of peaks and d is the number of SNPs recorded. X_{n*p} is the cells/groups/samples-by-peaks matrix from the Anndata object. The cell/group/samples by SNP matrix $S_{n*s} = X_{n*p}D_{p*s}$ is then found. For scCC data, cells from the sample cell type are added together. When plotted, S_{n*s} is divided by the total number of cells and normalized by a single SNP. Additionally, Pycallingcards also provides functionality to output all SNPs (and their associated annotations) that are under or near a set of user-specified peaks.

Connection to WashU Epigenome Browser

Pycallingcards provides functionality to visualize CC and peak data directly in the WashU Epigenome Browser (Li, Hsu, Purushotham, Sears, & Wang, 2019; Li et al., 2022). CC data is in qbed format(Moudgil et al., 2020) and TF directed insertions and a track displaying the density of insertions can be visualized in the Epigenome browser by

supplying files in qbed and bedgraph formats respectively. Pycallingcards provides the function WashU_browser_url to sort and compress and index these files. Pycallingcards then converts the density bedgraph file to bigwig format by UCSC Kent bedGraphToBigWig utility (Kent, Zweig, Barber, Hinrichs, & Karolchik, 2010). WashU Epigenome Browser handles all file processing and hosting and Pycallingcards generates a datahub.json for WashU Epigenome Browser and returns a 24h-valid link to WashU Epigenome Browser for all visualization. The codes are available on Github.

Bulk Calling Cards data Generation

In order to compare the performance of different methods, we generated a large Brd4 CC data collected from K562 cells.

In vitro bulk calling card experiments

The K562 cell line was cultured in RPMI medium containing 10% FBS and penicillin-streptomycin. 2×10^6 cells were transfected with 5ug of donor Barcoded-PBase-SRT-Puro and 5ug helper (hyPBase or hyPBase-centrip) using the Neon electroporation system for a total of 30 replicates. For a negative control, one replicate of K562 cells was transfected with 5ug Barcoded-PBase-SRT-Puro plasmid only. We used the following settings-pulse voltage: $1,450$ V; pulse width: 10 ms; pulse number: 3. After transfection, three replicates were plated into a 10 cm dish. Cells were grown under 2ug/ml puromycin selection for 11 days, by which time almost all negative control transfectants were dead. After 11 days, each replicate was dissociated into a single-cell suspension with 0.25% trypsin-EDTA and resuspended in PBase. Aliquots of each replicate were cryopreserved in culture media supplemented with 5% DMSO. The remaining cells were pelleted by centrifugation at 300 g for 5 minutes. Cell pellets were either processed immediately or kept at -80C in RNAProtect Cell Reagent.

Isolation and RT of bulk RNA

Total RNA was isolated from every three replicates using the RNEasy Plus Mini Kit following manufacturer's instructions and as described in ((Moudgil et al., 2020)). RNA was eluted in 40 ul RNase-free water and quantified using the Qubit RNA HS Assay Kit.

We performed first strand synthesis on each replicate with Maxima H Minus Reverse Transcriptase. We mixed 2μg of total RNA with $1\mu l of 10$mM dNTPs and 1μl of 50μM SMART_dT18VN primer, brought the total volume up to 14μl, and incubated it at 65°C for 5 minutes. After a 1 minute incubation on ice, we added 4μl X Maxima RT Buffer, 1μl RNaseOUT, and 1μl of Maxima H Minus Reverse Transcriptase. The solution was mixed by pipetting and incubated at 50°C for 1 hour followed by heat inactivation at 85°C for 10 minutes. cDNA was stored at -20°C.

Amplifying self-reporting transcripts from RNA

The PCR conditions for amplifying self-reporting transcripts (i.e., transcripts derived from self-reporting transposons) involved mixing 1μl cDNA template with 12.5μl Kapa HiFi HotStart ReadyMix, 0.5μl25μM SMART primer, and 1μl of 25μM SRT_PAC_F1 primer. The mixture was brought up to 25μl with ddH20. Thermocycling parameters were as follows: 95°C for 3 minutes; 20 cycles of: 98°C for 20 s $-$ 65°C for 30 s $-$ 72°C for 5 minutes; 72°C for 10 minutes; hold at 4C forever. As a control, cDNA quality can be assessed with exon-spanning primers for b-actin under the same thermocycling settings.

PCR products were purified using AMPure XP beads. 15μl(0.6X) resuspended beads were added to the 25ul PCR product and mixed homogenously by pipetting. After a 5 minute incubation at room temperature, the solution was placed on a magnetic rack for 2 minutes. The supernatant was aspirated and discarded. The pellet was washed twice with 200μl of 80% ethanol (incubated for 30 s each time), discarding the supernatant each time. The samples were briefly spun down and the tubes were placed against a magnetic rack; the final few drops of supernatant wer then removed with a p10 pipette. Next, 20μlddH20 was added to the beads and DNA was resuspended by pipetting. The slurry was incubated at room temperature for 2 minutes, and placed on a magnetic rack for one minute. Once clear, the supernatant was transferred to a clean 1.5 mL tube. DNA concentration was measured on the Qubit 3.0 Fluorometer using the dsDNA High Sensitivity Assay Kit.

Generation of bulk RNA calling card libraries

Calling card libraries from bulk RNA were generated using the Nextera XT DNA Library Preparation Kit. 1ng of PCR product was resuspended in 5μl nuclease-free water. To this mixture, we added 10μl Tagment DNA (TD) Buffer and 5μ l Amplicon Tagment Mix (ATM). After pipetting to mix, we incubated the solution in a thermocycler preheated to $55°$C. The tagmentation reaction was halted by adding 5μ l Neutralize Tagment (NT) Buffer and was kept at room temperature for 5 minutes. The final PCR was set up by adding 15μl Nextera PCR Mix (NPM), 8μl H20, 1μl of 10mM transposon primer (e.g., OM-PBase-NNN-Index2) and 1μl Nextera N7 index1 primer. The final PCR was run under the following conditions: $95°$C for 30 s; 13 cycles of: $95°$C for 10 s – $50°$C for 30 s – $72°$C for 30 s; $72°$C for 5 minutes; hold at $4°$C forever. After PCR, the final library was purified using 30μl$(0.6$x$)$ AMPure XP beads, as described above. The library was eluted in 11μlddH20 and quantitated on an Agilent TapeStation 4200 System using the High Sensitivity D1000 Screentape.

Sequencing and analysis

The librares were sequenced on an Illumina NovaSeq 6000.

Data analysis

A custom nf-core/callingcards pipeline nf-core/callingcards pipeline was built using Nextflow(?, ?), to provide a portable pipeline for the community. Briefly, the fully containerized pipeline takes in raw fastq files as input and parses the SRT barcodes, trims the adapters, aligns to a reference genome, and generates a resulting qBED file, which can be used directly as input into Pycallingcards.

To benchmark the peak calling performance, we conducted a benchmark test with a sample size ranging from 10,000 to 20 million insertions. This range was chosen as it reflects the scope of standard experiments, and it also accounts for the detection of weaker binding signals when a large number of insertions are gathered, necessitating the application of non-typical p-value thresholds.

Additionally, HOMER 4.11 is used for motif calling.

1 Notes

1.1 Results for Mouse Cortex Calling Cards data

Here we provide supplemental information about our analysis of the Mouse Cortex Calling Cards(CC) data. Table 1 shows the final results for mouse cortex data after preprocessing, differential peak analysis, pair analysis, and GWAS analysis. Each column contains information on the cluster, peak information, gene information, the locus that peaks mapped to hg38, the gene locus that mapped to hg38, and the GWAS published results of the mapped peak area. For example, the peak chr16:43501178...43518253 is significantly expressed in Astrocytes (Logfoldchange = 3.077262 and adjusted p-value = 3.297735e-14) and potentially regulates the gene Zbtb20 (score of 79.234276 and adjusted p-value = 0.000000e+00). In humans, Zbtb20 is encoded at hg38(chr3:114314499...115147280) and the binding peak is at hg38, chr3:114439800...114457200. In this genomic region, single nucleotide variants (SNVs) have been identified through GWAS as being significantly associated with several key phenotypes, including Schizophrenia, smoking status (distinguishing between ever and never smokers), smoking initiation, and vertex-wise sulcal depth. Further analysis of the GWAS database revealed distinct cell types exhibiting differentially bound peaks in proximity to these GWAS-associated SNVs (see Fig 1). Notably, a considerable number of SNVs, which show associations with intelligence-related outcomes such as cognitive ability, intelligence, attention deficit hyperactivity disorder (ADHD), and autism spectrum disorder — a phenomenon indicative of pleiotropy — are situated near neuron-specific Brd4 binding peaks. This analysis provides a testable hypothesis about a potentially crucial role of this variant in influencing Zbtb20 gene regulation which in turn could affect a diverse range of cognitive abilities and disorders.

1.2 Results for Bulk Glioblastoma Calling Cards data

Enhancers differentially bound by Brd4 in female and male mouse glioblastoma (GBM) cells(Kfoury et al., 2021) are shown in Table 3. A log fold change larger than 0 corresponds to increased Brd4 binding in female cells. Tables 4 and 5 report DNA motifs that were significantly enriched in male-bound or female-bound enhancers respectively. To find these motifs, differentially bound peaks were first filtered (adjusted p-value ≤ 0.05, log fold change ≥ 3) and then HOMER was utilized to call motifs separately. Motifs with p-value ≤ 0.05 are reported.

Motifs for three TF families were enriched in female-specific Brd4 peaks: IRF, ETS, MADS. The IRF family, particularly IRF5, has previously been implicated in mediating sex differences in immune responses. Higher levels of IRF5 in females were found to be correlated with increased production of specific cytokines like IFN-α and TNF-α, underlining the importance of considering sex differences in immune response studies and potential therapeutic approaches (Griesbeck et al., 2015; Beisel et al., 2023; Andrilenas et al., 2018). In addition, the interaction between the IRF (Interferon Regulatory Factor) motif family and Brd4 (Bromodomain-containing protein 4) is supported by a previous study investigating IRF8's role in regulating the transcription of Naip genes, which are critical for the activation of the NLRC4 inflammasome. This work showed that Brd4 collaborates with IRF8 and PU.1 to regulate gene transcription. ChIP-seq and qPCR analyses revealed the enrichment of Brd4, IRF8, PU.1, and RNAPII at the promoters of Naip2 and Naip5/6. The binding of Brd4 to these promoters is IRF8-dependent, and mutations in the IRF8 or PU.1 binding motifs disrupt the formation of these transcriptional complexes, underscoring the essential role of these motifs in recruiting Brd4 to the promoters to facilitate Naip gene transcription(Dong et al., 2021). These studies support the idea that IRF TFs are involved in establish sex differences in a Brd4 mediated manner, and our results suggest that further investigations into the role of these TFs in GBM is warranted. MADS-box genes have been implicated in plant sex determination (X. Zhang et al., 2023; Fatima et al., 2020), so there is some weak support in the literature for our observations. The ETS family has not previously been implicated in playing a role in sex differences. Further experimentation will be required to ascertain the roles of these two TF families in establishing sex differences(Grant, Wang, Kumari, Zabet, & Schalkwyk, 2022; Okada et al., 2011).

One motif of interest that was found to be enriched in male-specific Brd4 bound enhancers is the SRY motif. In mammals, particularly humans, the SRY (sex-determining region Y) transcription factor plays a crucial role in male sex determination by activating transcription of genes critical for male development (Weiss, 2005). Thus an intriguing possibility raised by these results is that a portion of the transcriptional differences between male and female GBM cells can be traced back to the sex-determining transcription factor SRY.

1.3 Results for yeast Calling Cards data

MACCs peak calling provides a more elegant method for analysis of Saccharomyces cerevisiae CC data that is independent of genome feature annotations. CC data for TFs in Saccharomyces cerevisiae has heretofore been analyzed by calculating a statistical enrichment of Ty5 insertions in intergenic regions, which were annotated as sequences between ORFs that are less than 5kb in length; this analysis ignored non-protein coding genes and other genomic features. This analysis method also gave no indication of binding peak size. Pycallingcards also offers additional functional features not previously available in yeast CC data analysis, such as the footprint function. Because the Ty5 transposon used in the yeast CC assay has no insertion sequence bias, binding targets with large numbers of insertions often display two peak summits that flank the TF's sequence motif, thought to be caused by steric hindrance of TF binding. The Pycallingcards footprint function can be used to identify this absence of insertions within the peak, which could be used as a tool to identify TF binding sites with greater resolution.

We next performed peak annotation and differential peak binding analysis of published yeast CC data. Here, we analyzed yeast CC data from Shively et al (Shively, Liu, Chen, Loell, & Mitra, 2019). Specifically, we analyzed the yeast TF Tye7p in wild type and *gcr2*Δ yeast, in which the coding sequence of the TF Gcr2p has been deleted from the genome. From these CC experiments and other data, Gcr2p and Tye7p were found to bind genomic targets together with Rap1p and Gcr1p as a cooperative collective. To identify Tye7p targets differentially bound in the presence or absence of Gcr2p, qbed files from Tye7p CC assays conducted in the two strains (WT vs *gcr2*Δ) were combined. MACCs was then used with a window size of 125, step size 50, and p-value cutoff of 1e-4 to call 68 peaks in total, with the Fisher's exact test used to calculate significant binding. Following peak calling, the annotation function was used to find the closest genes upstream and downstream of each peak. We then overlaid mRNA expression microarray data from either deletion of GCR2 or overexpression of Tye7p and Gcr2p Tye7p(Hackett et al., 2020; Kemmeren et al., 2014) to connect mRNA levels to Tye7p binding, since Tye7p and Gcr2p are thought to bind cooperatively. Table 6 shows the full results for differential binding of Tye7p between the WT and GCR2 deletion strain. Each row represents one peak and shows whether it is significantly more bound in the WT strain (group: Tye7p) or the *gcr2*Δ strain (group: Tye7p *gcr2*Δ). In agreement with previous work, Tye7p has significantly decreased binding to native targets in the absence of Gcr2p. Most of these MACCs-called peaks are associated with strong gene expression changes when Tye7p or another member of the cooperative collective is perturbed, according to mRNA expression signatures. Furthermore, we now report spurious Tye7p targets that appear in the absence of Gcr2p. Somewhat perplexingly, several of these new targets that appear upon deletion of Gcr2p may in fact be authentic Tye7p targets, as they are also associated with downstream gene expression changes. The differential Tye7p binding uncovered by MACCs in conjunction with Pycallingcards recapitulates previous results and provides new avenues for discovery of gene regulatory mechanisms.

2 Addition notes for peak calling

Given that the PBase transposase targets TTAA tetranucleotides with exclusivity, these sites inherently represent the natural potential for binding events across the genome. While the distribution of TTAA sites is random, it is not uniform, leading to differential insertion probabilities. To empirically demonstrate the necessity of accounting for TTAA sites in our analysis, we conducted peak calling on the same unfused data in Figure 2c left against a backdrop of uniform genomic distribution. As depicted in the accompanying Figure 4, the incorporation of TTAA site consideration significantly enhances the congruence of our peak calling results with the ChIP-seq data signals. This clearly indicates that integrating TTAA site prevalence into our peak calling methodology is crucial for accurately capturing the true signal intensity and distribution.

2.1 Simulation

To more effectively demonstrate our methods' feasibility, we performed simulation analyses on both fused and unfused data in bulk and single-cell scenarios.

In the unfused simulation, our initial step involved analyzing the interaction between ChIP-seq H3K27ac (ENCFF044JNJ) and Brd4 (ENCFF130JVF) data of K562 cell line sourced from ENCODE. We then extended the peak lengths and applied filters based on the presence of TTAA sites. The data was presumed to include: 1) insertions from authentic Brd4 peaks, 2) insertions from random peaks, and 3) random insertions across the genome. Notably, the number of insertions from actual Brd4 peaks outnumbered those from random peaks by 28.9 times. These random peaks were uniformly distributed throughout the genome, avoiding overlap with genuine Brd4 peaks(Gogol-Döring et al., 2016).

In the SP1 data simulation, we treated CHIP-seq peaks downloaded from ENCODE (ENCFF044JNJ) similarly, extending and filtering them. A critical point to consider is the potential overlap between SP1 and Brd4 peaks. Insertions from SP1 peaks were 100 times more frequent than those from random peaks. The bulk dataset comprised over ten million insertions, while the single-cell dataset contained approximately two million.

Three key metrics were charted (Figure 5): sensitivity, specificity, and False Discovery Rate (FDR). True Positivity (TP) was defined as the occurrence of genuine peaks overlapping by at least 200 base pairs (bp). False Positivity (FP) was determined by the count of random peaks in unfused data or the aggregate of random peaks and non-overlapping Brd4 and SP1 peaks. True Negativity (TN) was calculated as the total false peaks minus FP, and False Negativity (FN) as the total real peaks minus TP.

Sensitivity, or the true positive rate, gauges the proportion of correctly identified actual positives. It is the ratio of TP (real peaks overlapping with called peaks by at least 200 bp) to the sum of TP and FN (real peaks not overlapping with called peaks). Specificity, or the true negative rate, evaluates the proportion of accurately identified actual negatives, calculated as the ratio of TN (total false peaks minus FP) to the sum of TN and FP (called peaks not corresponding to real peaks or Brd4 peaks not overlapping with SP1 peaks).

A peak qualifies as genuine based on its overlap with real peaks, provided the overlap is at least equal to that of false peaks. The FDR is determined as 1 minus the ratio of genuine peaks identified among all peaks.

Our results demonstrate that CCcaller, MACCs and ccf_tools exhibit superior sensitivity and specificity compared to other methods. Notably, the peaks identified are highly specific, resulting in an exceptionally low false positive rate. In summary, CCcaller is recommended for unfused data, whereas MACCs may be more suitable for fused data.

3 Figures

3.1 Figure 1

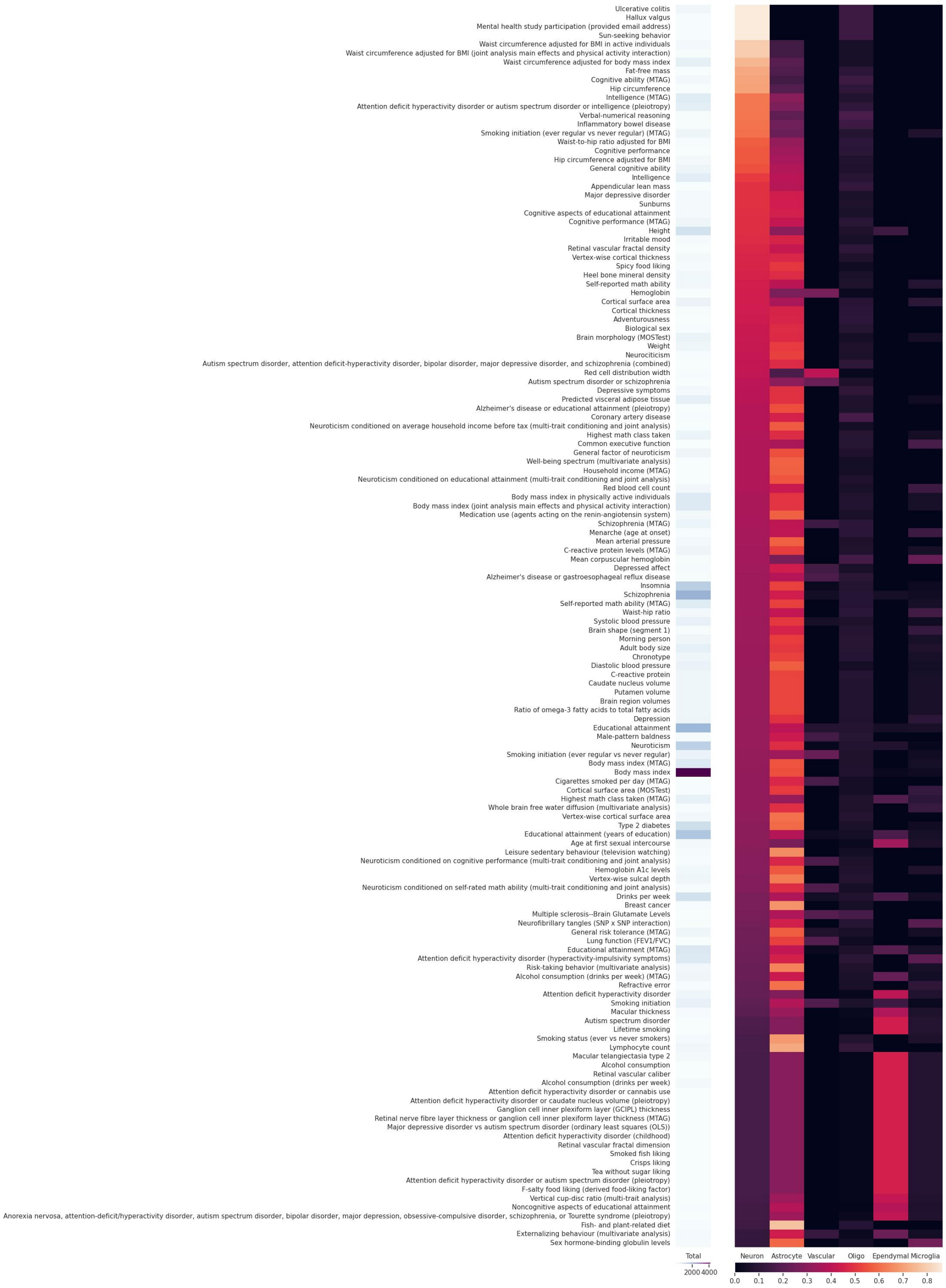

Figure 1: GWAS results for mouse cortex data among different cell types. The first column indicates the total times detected. The rest six columns show the relative time detected by different cell types.

3.2 Figure 2

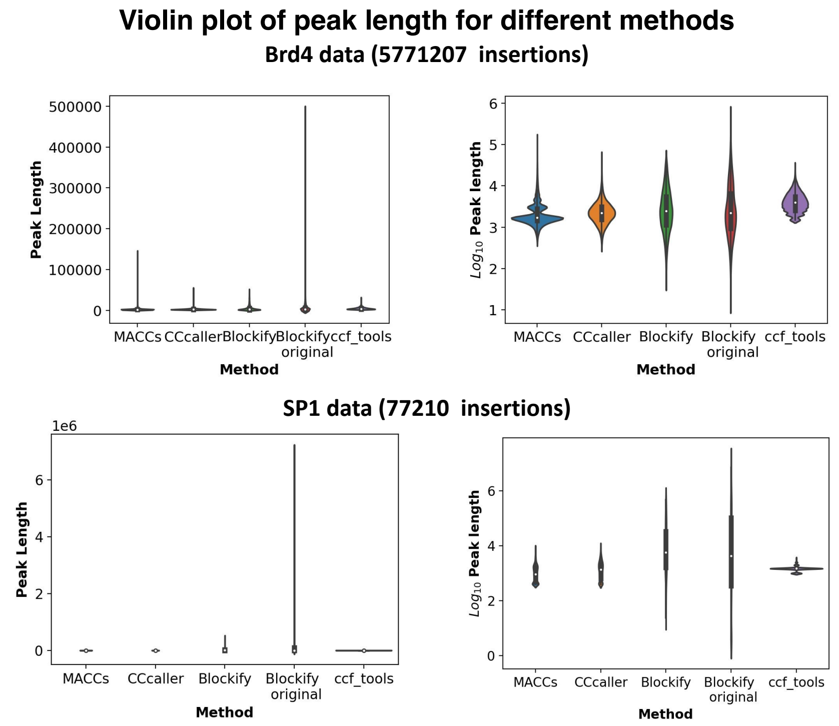

Figure 2: Violin plot of peak length for different methods with Brd4 data and SP1 data.

3.3 Figure 3

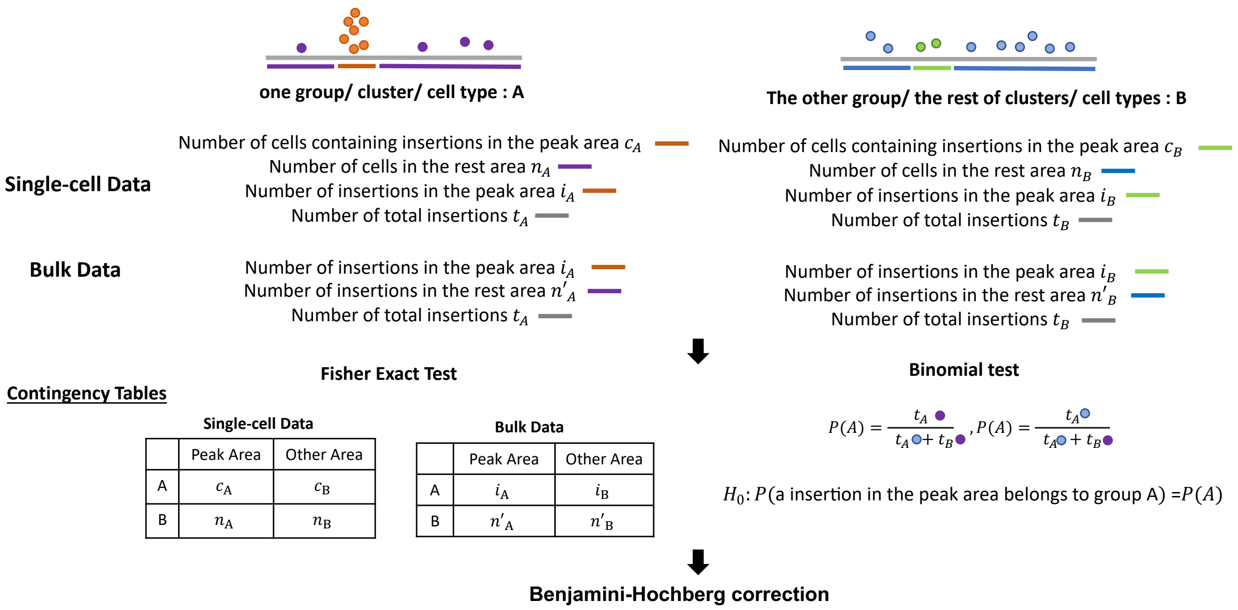

Figure 3: Differential peak analysis workflow.

3.4 Figure 4

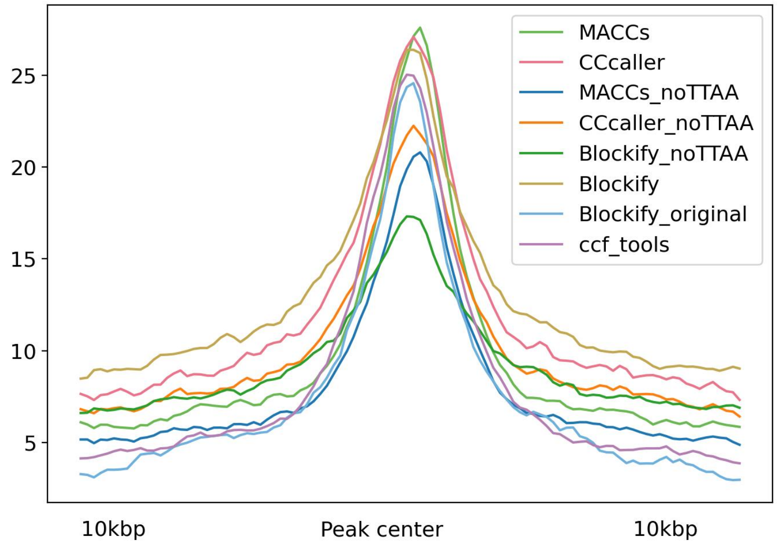

Figure 4: Chip-seq signal in calling cards peaks for the different peak calling methods in Pycallingcards: Comparing with/without TTAA distribution

3.5 Figure 5

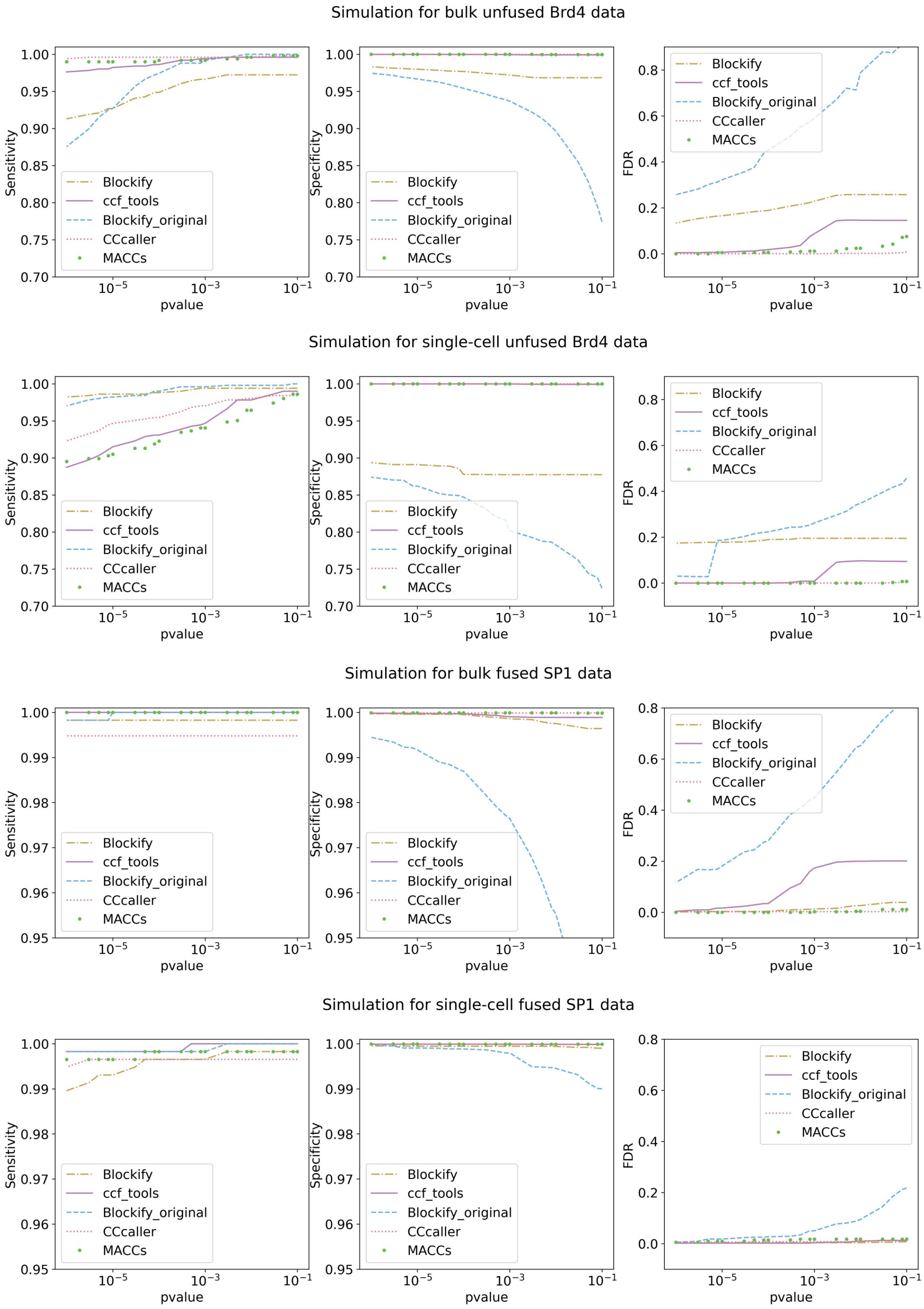

Figure 5: Simulation result.

4 Tables

4.1 1

Table 1: Data Resources

DATASET	SOURCE	IDENTIFIER
HyPBase calling cards data in K562 cell line	This study	GEO: GSE248420
HyPBase calling cards data in HCT116 cell line	Robi D. Mitra<br>(Moudgil et al., 2020)	GEO: GSM4471638
SP1 hyPBase calling cards data in HCT116 cell line	Robi D. Mitra<br>(Moudgil et al., 2020)	GEO: GSM4471639
HyPBase single-cell calling cards data in HCT116 cell line	Robi D. Mitra<br>(Moudgil et al., 2020)	GEO: GSM4471646
SP1 hyPBase single-cell calling cards datain HCT116 cell line	Robi D. Mitra<br>(Moudgil et al., 2020)	GEO: GSM4471648
ChIP-seq H3K27ac data in HCT116 cell line	Mark Gerstein<br>(J. Zhang, Lee, et al., 2020)	ENCODE:<br>ENCSR661KMA<br>(ENCFF997CJQ)
ChIP-seq SP1 data in HCT116 cell line	ENCODE<br>(Consortium et al., 2012)	ENCODE:<br>ENCFF587ZMX
Mouse cortex single-cell hyPBase calling cards data	Robi D. Mitra<br>(Moudgil et al., 2020)	GEO: GSM4471660
Mouse cortex single-cell hyPBase RNA-seq data	Robi D. Mitra<br>(Moudgil et al., 2020)	GEO: GSM4471659
Brd4-bound enhancers drive cell intrinsic calling cards data in sex differences of glioblastoma	Joshua B. Rubin<br>(Kfoury et al., 2021)	GEO: GSE156678
Brd4-bound enhancers drive cell intrinsic RNA-seq data in sex differences of glioblastoma	Joshua B. Rubin<br>(Kfoury et al., 2021)	GEO: GSE156819
Tye7p yeast calling cards data	Robi D. Mitra<br>(Shively et al., 2019)	GEO: GSM3946397
Tye7p in Δ strain background yeast calling cards data	Robi D. Mitra<br>(Shively et al., 2019)	GEO: GSM3946398
Compendium of deletion mutant gene expression profiles for *gcr2*Δ deletion	Frank C.P. Holstege<br>(Kemmeren et al., 2014)	GEO: GSE42527
A small molecule transcriptional induction system is used to induce TFs in Saccharomyces cerevisiae	R Scott McIsaac<br>(Hackett et al., 2020)	GEO: GSE142864
ChIP-seq H3K27ac data in K562 cell line	Mark Gerstein<br>(J. Zhang, Lee, et al., 2020)	ENCODE:<br>ENCSR000AKP<br>(ENCFF044JNJ)
ChIP-seq Brd4 data in K562 cell line	Mark Gerstein<br>(J. Zhang, Liu, et al., 2020)	ENCODE:<br>ENCSR583ACG<br>(ENCFF130JVF)

ChIP-seq SP1 data in K562 cell line	ENCODE<br>(Consortium et al., 2012)	ENCODE:<br>ENCSR000BKO<br>(ENCFF171NEU)

4.2 2

Table2: Differentiated Results for Mouse Cortex Data

Cluster	Peak	Logfold-changes for Peak	Pvalue_peak	Pvalue_adj_peak	Gene	Score_gene	Pvalue_gene	Pvalue_adj_gene	Chr_liftover	Start_liftover	End_liftover	Chr_hg38	Start_hg38	End_hg38	GWAS
Astrocyte	chr16_43501178_43518253	3.077262	1.462410e-16	3.297735e-14	Zbtb20	79.234276	0.000000e+00	0.000000e+00	chr3	114439800	114457200	chr3	114314499	115147280	Schizophrenia; Smoking status (ever vs never smokers); Smoking initiation; Vertex-wise sulcal depth
Astrocyte	chr8_64645834_64659215	4.623955	3.114365e-14	3.794710e-12	Msmo1	61.964111	0.000000e+00	0.000000e+00	chr4	165418445	165438029	chr4	165327665	165343162	Atopic dermatitis (moderate to severe)
Astrocyte	chr3_141928409_141939733	4.876938	3.786296e-14	3.794710e-12	Bmpr1b	24.131538	2.475603e-121	1.659753e-120	chr4	95040417	95054959	chr4	94757976	95158450	
Astrocyte	chr4_97575305_97588788	3.099679	5.907086e-14	5.328192e-12	E130114P18Rik	16.893749	3.380841e-62	1.514206e-61	chr1	60858298	60872361				Refractive error
Astrocyte	chr4_97575305_97588788	3.099679	5.907086e-14	5.328192e-12	Nfia	45.417362	0.000000e+00	0.000000e+00	chr1	60858298	60872361	chr1	61077273	61462788	Refractive error
Astrocyte	chr6_36787352_36796453	4.732581	1.169436e-12	8.114085e-11	Ptn	54.479534	0.000000e+00	0.000000e+00	chr7	137319476	137327945	chr7	137227345	137343990	Educational attainment (years of education)
Astrocyte	chr19_55100686_55103496	11.581732	3.142675e-12	2.024780e-10	Gpam	16.416140	6.481936e-59	2.812270e-58	chr10	112188868	112189882	chr10	112149863	112183779	
Astrocyte	chr7_54834211_54844532	4.164636	6.770998e-10	2.655409e-08	Siglech	-17.794437	1.929032e-70	9.217479e-70	chr11	24495239	24507621				Plasma neurofilament light levels
Astrocyte	chr7_54834211_54844532	4.164636	6.770998e-10	2.655409e-08	Luzp2	59.469765	0.000000e+00	0.000000e+00	chr11	24495239	24507621	chr11	24496969	25082640	Plasma neurofilament light levels
Astrocyte	chr8_89307714_89312307	5.176061	1.618619e-09	5.615364e-08	Sall1	15.833781	4.203102e-55	1.750790e-54	chr16	51439323	51443812	chr16	51135974	51151272	
Astrocyte	chr15_95649630_95653550	5.176061	1.618619e-09	5.615364e-08	Dbx2	38.563210	3.149195e-282	4.054453e-281	chr12	45045820	45049965	chr12	45014755	45051099	
Astrocyte	chr6_141524183_141528573	11.096493	6.078344e-09	1.890575e-07	Slco1c1	49.340008	0.000000e+00	0.000000e+00	chr12	20695270	20698151	chr12	20695354	20753386	
Astrocyte	chr1_14302176_14310895	5.916935	9.478474e-09	2.757930e-07	Eya1	25.117405	8.534710e-131	6.012811e-130	chr8	71354173	71362942	chr8	71197432	71362232	Chronic obstructive pulmonary disease-related biomarkers
Astrocyte	chr4_105166110_105175360	5.916935	9.478474e-09	2.757930e-07	Plpp3	162.559265	0.000000e+00	0.000000e+00	chr1	56558059	56567379	chr1	56494746	56579584	
Astrocyte	chr13_89583161_89588793	4.356304	2.136393e-07	4.817567e-06	Hapln1	34.593361	9.429821e-234	1.012643e-232	chr5	83666573	83675163	chr5	83638197	83721077	Vaginal microbiome MetaCyc pathway (PYRIDNUCSYN-PWY|NAD biosynthesis I (from aspartate)); Vaginal microbiome MetaCyc pathway (PWY-2941|L-lysine biosynthesis II); Vaginal microbiome MetaCyc pathway (PWY-2942|L-lysine biosynthesis III); Vaginal microbiome MetaCyc pathway (VALSYN-PWY|L-valine biosynthesis)
Astrocyte	chr6_36774153_36779449	3.603368	2.292117e-07	4.922594e-06	Ptn	54.479534	0.000000e+00	0.000000e+00	chr7	137302397	137307872	chr7	137227345	137343990	
Astrocyte	chr2_170286475_170289090	4.797702	2.987844e-07	5.390070e-06	Bcas1	-25.416523	2.540125e-139	1.862290e-138	chr20	53865018	53867907	chr20	53943537	54070765	
Astrocyte	chr13_119753053_119756787	4.797702	2.987844e-07	5.390070e-06	Nim1k	25.919909	7.377848e-139	5.398411e-138	chr5	43191219	43195768	chr5	43192067	43280850	
Astrocyte	chr2_83826634_83830409	3.749735	4.048661e-07	6.639805e-06	Itgav	22.455799	1.326365e-106	8.246725e-106	chr2	186711540	186714686	chr2	186590062	186680902	Inflammatory bowel disease; Crohn’s disease
Astrocyte	chr3_34660305_34666432	3.749735	4.048661e-07	6.639805e-06	Sox2	48.070873	0.000000e+00	0.000000e+00	chr3	181722645	181728925	chr3	181711923	181714435	Diastolic blood pressure (baseline)
Astrocyte	chr4_97842250_97846042	3.487933	1.046381e-06	1.573060e-05	E130114P18Rik	16.893749	3.380841e-62	1.514206e-61	chr1	61154080	61158476				Thyroid hormone levels; Erectile dysfunction
Astrocyte	chr4_97842250_97846042	3.487933	1.046381e-06	1.573060e-05	Nfia	45.417362	0.000000e+00	0.000000e+00	chr1	61154080	61158476	chr1	61077273	61462788	Thyroid hormone levels; Erectile dysfunction
Astrocyte	chr4_97757056_97761515	3.189871	1.273742e-06	1.853089e-05	Nfia	45.417362	0.000000e+00	0.000000e+00	chr1	61056667	61063071	chr1	61077273	61462788	
Astrocyte	chr1_161271808_161275172	4.645772	1.656509e-06	2.334642e-05	Prdx6	110.561226	0.000000e+00	0.000000e+00	chr1	173430714	173438520	chr1	173477346	173488807	
Astrocyte	chrX_66657396_66661253	4.645772	1.656509e-06	2.334642e-05	Slitrk2	23.033348	1.539695e-111	9.809484e-111	chrX	145825915	145829852	chrX	145817828	145825842	
Astrocyte	chr15_95690995_95691836	10.582202	1.772759e-06	2.460044e-05	Dbx2	38.563210	3.149195e-282	4.054453e-281	chr12	45089094	45089934	chr12	45014755	45051099	
Astrocyte	chr18_81165183_81167067	5.457728	2.083387e-06	2.684593e-05	Sall3	27.820368	1.091092e-157	8.722163e-157	chr18	78701389	78703287				
Astrocyte	chr4_97902048_97910959	3.362452	4.659168e-06	5.756945e-05	E130114P18Rik	16.893749	3.380841e-62	1.514206e-61	chr1	61218524	61226920				Triglyceride levels; Sex hormone-binding globulin levels adjusted for BMI; Sex hormone-binding globulin levels; Total testosterone levels; Non-HDL cholesterol levels; Hair color
Astrocyte	chr4_97902048_97910959	3.362452	4.659168e-06	5.756945e-05	Nfia	45.417362	0.000000e+00	0.000000e+00	chr1	61218524	61226920	chr1	61077273	61462788	Triglyceride levels; Sex hormone-binding globulin levels adjusted for BMI; Sex hormone-binding globulin levels; Total testosterone levels; Non-HDL cholesterol levels; Hair color
Astrocyte	chr16_43462811_43467699	4.066931	5.729681e-06	6.890897e-05	Zbtb20	79.234276	0.000000e+00	0.000000e+00	chr3	114495525	114500568	chr3	114314499	115147280	Height
Astrocyte	chr7_93061766_93064465	5.265201	1.231085e-05	1.321950e-04	Fam181b	37.657539	4.819317e-271	5.941159e-270	chr11	82754461	82758070	chr11	82732003	82733864	
Astrocyte	chr12_53947288_53948638	10.359967	1.175631e-05	1.321950e-04	Npas3	53.549488	0.000000e+00	0.000000e+00	chr14	33676336	33677661	chr14	32939252	33804176	
Astrocyte	chr4_43971688_43975452	5.265201	1.231085e-05	1.321950e-04	Glipr2	9.467184	4.420477e-21	1.157486e-20	chr9	36151753	36155167	chr9	36136535	36163913	
Astrocyte	chr4_132514332_132516335	5.265201	1.231085e-05	1.321950e-04	Sesn2	9.228589	3.980041e-20	1.019879e-19	chr1	28247139	28249353	chr1	28259451	28282491	
Astrocyte	chr14_103833967_103838400	10.359967	1.175631e-05	1.321950e-04	Ednrb	56.828690	0.000000e+00	0.000000e+00	chr13	77909156	77913819	chr13	77895480	77975723	
Astrocyte	chr6_141567438_141569615	3.897097	2.866836e-05	2.810745e-04	Slco1c1	49.340008	0.000000e+00	0.000000e+00	chr12	20750659	20752611	chr12	20695354	20753386	Ceramide (d17:1/16:0) levels
Astrocyte	chr6_141567438_141569615	3.897097	2.866836e-05	2.810745e-04	Slco1b2	9.574473	1.624957e-21	4.295453e-21	chr12	20750659	20752611				Ceramide (d17:1/16:0) levels
Astrocyte	chr5_9551451_9558364	3.897097	2.866836e-05	2.810745e-04	Grm3	25.906481	8.773381e-139	6.414478e-138	chr7	86792861	86810903	chr7	86643913	86864876	Schizophrenia; Schizophrenia vs ADHD (ordinary least squares (OLS)); Autism spectrum disorder or schizophrenia; Bipolar disorder (MTAG); Schizophrenia (MTAG); Anorexia nervosa, attention-deficit/hyperactivity disorder, autism spectrum disorder, bipolar disorder, major depression, obsessive-compulsive disorder, schizophrenia, or Tourette syndrome (pleiotropy); Cognitive ability, years of educational attainment or schizophrenia (pleiotropy)
Astrocyte	chr10_57785182_57787861	5.042965	7.165764e-05	6.214923e-04	Smpdl3a	24.626255	4.559749e-126	3.142100e-125	chr6	122779971	122783477	chr6	122789048	122809719	
Astrocyte	chr10_57785182_57787861	5.042965	7.165764e-05	6.214923e-04	Fabp7	40.557236	9.333561e-309	1.296925e-307	chr6	122779971	122783477	chr6	122749200	122784077	
Astrocyte	chr19_18887469_18888841	5.042965	7.165764e-05	6.214923e-04	Rorb	39.974609	2.998225e-306	4.132123e-305	chr9	74728437	74729816	chr9	74497335	74687201	
Astrocyte	chrX_110808176_110813229	5.042965	7.165764e-05	6.214923e-04	Pou3f4	17.160261	4.431524e-64	2.016876e-63	chrX	83501946	83507160	chrX	83508260	83509767	
Astrocyte	chr10_56837049_56842478	3.704570	1.393020e-04	1.092612e-03	Gja1	161.039825	0.000000e+00	0.000000e+00	chr6	121845842	121851588	chr6	121435576	121449744	Resting heart rate
Astrocyte	chr12_25097345_25101520	3.704570	1.393020e-04	1.092612e-03	Id2	53.476070	0.000000e+00	0.000000e+00	chr2	8676341	8680475	chr2	8681982	8684453	Cognitive function (immediate memory) (longitudinal); Smoking initiation
Astrocyte	chr12_52471736_52476169	3.704570	1.393020e-04	1.092612e-03	Arhgap5	33.806904	2.084607e-226	2.184311e-225	chr14	32040797	32045534	chr14	32077288	32159728	
Astrocyte	chrX_82814085_82817872	3.704570	1.393020e-04	1.092612e-03	Dmd	22.847645	3.553036e-110	2.248239e-109	chrX	33336368	33340186	chrX	31119227	33339609	

Astrocyte	chr8_89034093_89040778	3.292603	3.329108e-04	2.345981e-03	Sall1	15.833781	4.203102e-55	1.750790e-54	chr16	51142875	51149485	chr16	51135974	51151272	Vertical cup-disc ratio (adjusted for vertical disc diameter); Vertical cup-disc ratio (multi-trait analysis); Insomnia; Vertex-wise sulcal depth; Self-reported math ability; Self-reported math ability (MTAG)
Astrocyte	chr3_121721058_121723454	4.780151	4.090009e-04	2.692838e-03	F3	63.434364	0.000000e+00	0.000000e+00	chr1	94541919	94545222	chr1	94529175	94541857	
Astrocyte	chrX_16909454_16912173	4.780151	4.090009e-04	2.692838e-03	Ndp	20.796898	6.535406e-92	3.707322e-91	chrX	43971374	43974093	chrX	43948775	43973675	
Astrocyte	chrX_16909454_16912173	4.780151	4.090009e-04	2.692838e-03	Maob	19.810839	6.363952e-84	3.394473e-83	chrX	43971374	43974093	chrX	43766609	43882475	
Astrocyte	chr14_34501484_34506018	4.780151	4.090009e-04	2.692838e-03	Bmpr1a	17.371780	9.519241e-66	4.383948e-65	chr10	86752934	86757882	chr10	86756638	86925188	
Astrocyte	chr9_13581428_13583991	4.780151	4.090009e-04	2.692838e-03	Maml2	13.798990	1.635705e-42	5.967817e-42	chr11	96137061	96139025	chr11	95976592	96343180	
Astrocyte	chr11_35976787_35978806	4.780151	4.090009e-04	2.692838e-03	Tenm2	-99.841782	0.000000e+00	0.000000e+00	chr5	168293642	168295706	chr5	167284837	168264157	
Astrocyte	chr4_109789254_109793743	3.482334	6.528802e-04	3.952335e-03	Cdkn2c	7.564842	4.603788e-14	1.029604e-13	chr1	50656621	50662354	chr1	50968694	50974637	Lymphocyte count; Height
Astrocyte	chr7_116029926_116034387	3.798360	1.252359e-03	6.930231e-03	Sox6	17.668941	9.259437e-68	4.326505e-67	chr11	16603094	16607614	chr11	15966448	16476388	White blood cell count
Astrocyte	chr12_90738459_90740320	3.798360	1.252359e-03	6.930231e-03	Dio2	48.128227	0.000000e+00	0.000000e+00	chr14	80211649	80213350	chr14	80197524	80231054	
Astrocyte	chr12_90738459_90740320	3.798360	1.252359e-03	6.930231e-03	Cep128	10.048061	1.585817e-23	4.353672e-23	chr14	80211649	80213350	chr14	80496477	80939540	
Astrocyte	chr19_18902266_18902906	3.798360	1.252359e-03	6.930231e-03	Rorb	39.974609	2.998225e-306	4.132123e-305	chr9	74711074	74711715	chr9	74497335	74687201	
Astrocyte	chr1_55225610_55226866	3.798360	1.252359e-03	6.930231e-03	Rftn2	20.722839	2.299958e-91	1.298347e-90	chr2	197674793	197676083	chr2	197570802	197675860	Body fat distribution (arm fat ratio); Body fat distribution (trunk fat ratio)
Astrocyte	chr12_53914480_53917497	3.798360	1.252359e-03	6.930231e-03	Npas3	53.549488	0.000000e+00	0.000000e+00	chr14	33640894	33643982	chr14	32939252	33804176	
Astrocyte	chr4_154546502_154550898	3.798360	1.252359e-03	6.930231e-03	Prdm16	15.261837	2.250185e-51	9.028050e-51	chr1	3160800	3166648	chr1	3069177	3438621	Migraine; Principal component-derived dietary pattern 4
Astrocyte	chr14_31780804_31782401	3.070367	1.423248e-03	7.596268e-03	Btd	11.012485	7.095636e-28	2.114577e-27	chr3	15797070	15798633	chr3	15601351	15647634	
Astrocyte	chr12_53298464_53301782	4.458552	2.273285e-03	1.160791e-02	Npas3	53.549488	0.000000e+00	0.000000e+00	chr14	32993417	32999226	chr14	32939252	33804176	
Astrocyte	chr8_94153018_94155646	3.219519	2.923460e-03	1.425384e-02	Mt3	156.531128	0.000000e+00	0.000000e+00	chr16	56589862	56592351	chr16	56589354	56591088	
Astrocyte	chr3_121705823_121707208	3.219519	2.923460e-03	1.425384e-02	F3	63.434364	0.000000e+00	0.000000e+00	chr1	94584342	94585840	chr1	94529175	94541857	
Astrocyte	chr6_22908664_22910715	3.476762	6.001854e-03	2.666834e-02	Aass	12.359412	1.525315e-34	5.022563e-34	chr7	121913216	121915571	chr7	122073543	122144290	
Astrocyte	chr6_22908664_22910715	3.476762	6.001854e-03	2.666834e-02	Ptprz1	243.727875	0.000000e+00	0.000000e+00	chr7	121913216	121915571	chr7	121873104	122062036	
Astrocyte	chr2_83833412_83837135	3.476762	6.001854e-03	2.666834e-02	Itgav	22.455799	1.326365e-106	8.246725e-106	chr2	186718025	186721397	chr2	186590062	186680902	
Astrocyte	chr19_18873177_18876012	3.476762	6.001854e-03	2.666834e-02	Rorb	39.974609	2.998225e-306	4.132123e-305	chr9	74739831	74743266	chr9	74497335	74687201	Cortical surface area; Vertex-wise cortical surface area
Neuroblast_SVZ	chr18_23491771_23501860	4.033839	1.844968e-04	4.160403e-02	Dtna	-9.984835	5.373971e-21	3.447432e-20	chr18	34703602	34715659	chr18	34493289	34891844	
Neuron_Cajal-Retzius	chr4_109757395_109764176	5.972684	1.012198e-09	9.130028e-07	Cdkn2c	-16.580017	1.835613e-61	4.655913e-60	chr1	50699140	50706074	chr1	50968694	50974637	
Neuron_Cajal-Retzius	chr7_70361940_70375137	4.168978	1.658746e-04	2.234425e-02	Nr2f2	27.301947	1.630853e-104	1.063087e-102	chr15	96317885	96330499	chr15	96325927	96340263	Oral cavity cancer; Externalizing behaviour (multivariate analysis); Oropharynx cancer and human papilloma virus 16 negative oropharyngeal cancer; Smoking initiation
Neuron_Cajal-Retzius	chr4_109983536_109986912	5.331851	1.981752e-04	2.234425e-02	Dmrta2	10.020035	8.040660e-22	6.343073e-21	chr1	50413992	50417752	chr1	50417550	50423447	Educational attainment
Neuron_Excit_AON	chr2_61648111_61670758	3.168187	3.490750e-14	3.148657e-11	Tank	4.565320	5.350876e-06	1.690334e-05	chr2	161229841	161254167	chr2	161136954	161236172	Educational attainment (years of education); Educational attainment; Intelligence (MTAG); Cognitive ability (MTAG); Leisure sedentary behaviour (television watching); Major depressive disorder; Cognitive aspects of educational attainment; Smoking initiation (ever regular vs never regular) (MTAG); Cognitive traits (MTAG); Intelligence; Age at first sexual intercourse; Verbal-numerical reasoning; Brain morphology (MOSTest); Smoking initiation (ever regular vs never regular); Attention deficit hyperactivity disorder or autism spectrum disorder or intelligence (pleiotropy); Educational attainment (MTAG); Cognitive performance; Drinks per week; Cognitive performance (MTAG); Insomnia; Self-reported math ability; Self-reported math ability (MTAG); Highest math class taken (MTAG); Highest math class taken; Sunburns
Neuron_Excit_AON	chr3_18048625_18054529	4.654750	1.911138e-04	2.790018e-02	Cyp7b1	-7.446532	1.413722e-13	7.968485e-13	chr8	64574296	64580493	chr8	64586592	64798791	Automobile speeding propensity
Neuron_Excit_L2-4	chr1_41761840_41767895	3.282111	2.696358e-06	3.040144e-04	Pantr1	14.921903	6.755967e-50	3.801101e-49	chr2	103894021	103897184				Educational attainment
Neuron_Excit_L2-4	chr13_83141353_83148478	3.495487	3.141216e-06	3.148197e-04	Mef2c	182.292236	0.000000e+00	0.000000e+00	chr5	89235156	89249323	chr5	88718240	88904105	Macular thickness; Waist circumference adjusted for body mass index
Neuron_Excit_L5_Mixed	chr7_66014128_66014532	3.737143	2.786909e-04	3.677499e-02	Pcsk6	-16.587570	2.263189e-61	3.313281e-60	chr15	101337048	101337565	chr15	101303927	101489984	
Neuron_Granule_DG	chr3_125405334_125410657	5.806064	1.011677e-04	1.380304e-02	Ugt8a	-23.040115	5.201659e-89	3.379286e-87	chr4	115106106	115112630				
Neuron_Granule_DG	chr3_125405334_125410657	5.806064	1.011677e-04	1.380304e-02	Ndst4	4.497158	8.449939e-06	3.032315e-05	chr4	115106106	115112630	chr4	114827772	115113876	
Neuron_Granule_DG	chr1_50860999_50861403	4.356917	1.071190e-04	1.380304e-02	Tmeff2	3.399986	7.231398e-04	1.952972e-03				chr2	191949045	192194933	

The columns are: cluster (cell type), peak name, logfoldchange of peaks, p-value of differential binding analysis, adjusted p-value of differential binding analysis, the paired gene name, score of differential gene analysis, p-value of differential gene analysis, adjusted p-value of differential gene analysis, human chromosome number after ”lifting over” the peak to hg38, human start site after ”lifting over” the peak to hg38, human end site after ”lifting over” the peak to hg38, human chromosome number after ”lifting over” the gene to hg38, human start site after ”lifting over” the gene to hg38, human end site after ”lifting over” the gene to hg38, GWAS information within the peak areas after ”lifting over” the peak to hg38.

4.3 3

Table3: Differentiated Results for GBM Data

Peak	logfold-changes_peak	Pvalue_peak	Pvalue_adj_peak	Gene	Score_gene	Pvalue_gene	Pvalue_adj_gene	Chr_liftover	Start_liftover	End_liftover	Chr_hg38	Start_hg38	End_hg38	GWAS
chr1_174659622_174662521	5.652435	0.000000	0.000000	Grem2	-10.401831	0.000000	0.000000	chr1	240280756	240283835	chr1	240489572	240612162	Coronary heart disease
chr1_174917639_174921559	6.208699	0.000000	0.000000	Grem2	-10.401831	0.000000	0.000000	chr1	240608636	240611870	chr1	240489572	240612162	
chr2_93645657_93648162	5.189615	0.000002	0.000043	Alx4	-10.292504	0.000000	0.000000	chr11	44303696	44306415	chr11	44260727	44310166	
chr3_33140350_33142508	5.534632	0.000000	0.000002	Pex5l	-7.590236	0.000000	0.000000	chr3	180033883	180036186	chr3	179794958	180037053	
chr3_126498429_126499612	5.337659	0.000001	0.000012	Arsj	-6.281259	0.000000	0.000000	chr4	113839066	113844568	chr4	113900283	113979722	
chr3_126657669_126660188	-5.397757	0.000001	0.000012	Arsj	-6.281259	0.000000	0.000000	chr4	113683844	113686758	chr4	113900283	113979722	
chr3_132085669_132092999	6.121213	0.000000	0.000000	Dkk2	-6.916087	0.000000	0.000000	chr4	107029387	107035946	chr4	106921801	107036296	
chr3_132093815_132106295	5.072335	0.000000	0.000000	Dkk2	-6.916087	0.000000	0.000000	chr4	107015456	107028826	chr4	106921801	107036296	
chr3_141699368_141702608	6.065565	0.000000	0.000000	Bmpr1b	-5.570116	0.000000	0.000000	chr4	95306647	95309431	chr4	94757976	95158450	
chr4_68535893_68538867	5.172571	0.000000	0.000000	Brinp1	-7.758307	0.000000	0.000000	chr9	118993297	118996641	chr9	119166629	119369461	Gut microbiota (bacterial taxa, hurdle binary method)
chr4_68545353_68551019	6.825275	0.000000	0.000000	Brinp1	-7.758307	0.000000	0.000000	chr9	119002823	119008509	chr9	119166629	119369461	
chr4_118022206_118024522	-5.332271	0.000001	0.000022	Artn	9.083650	0.000000	0.000000	chr1	43824383	43825212	chr1	43933319	43937240	
chr5_13001869_13003460	5.957213	0.000000	0.000000	Sema3a	-5.771471	0.000000	0.000000				chr7	83958342	84194901	
chr5_13001869_13003460	5.957213	0.000000	0.000000	Sema3a	-5.771471	0.000000	0.000000				chr7	83958342	84194901	
chr5_13276479_13283560	7.727895	0.000000	0.000000	Sema3a	-5.771471	0.000000	0.000000	chr7	84314176	84318280	chr7	83958342	84194901	
chr5_13276479_13283560	7.727895	0.000000	0.000000	Sema3a	-5.771471	0.000000	0.000000	chr7	84314176	84318280	chr7	83958342	84194901	
chr5_17080302_17082934	5.024623	0.000010	0.000156	Hgf	-7.513756	0.000000	0.000000	chr7	81335127	81335733	chr7	81699007	81770198	
chr5_28465271_28467276	-5.397757	0.000001	0.000012	Shh	5.565109	0.000000	0.000000	chr7	155810616	155812624	chr7	155799983	155812273	Urate levels
chr5_38081825_38086331	6.088429	0.000000	0.000000	Nsg1	-5.086635	0.000000	0.000000	chr4	4488485	4493470	chr4	4386255	4419058	
chr7_56523378_56528436	6.721266	0.000000	0.000000	Gabrg3	-5.960560	0.000023	0.000132				chr15	26971281	27533227	
chr7_111875691_111878313	5.109476	0.000005	0.000082	Galnt18	-5.959958	0.000000	0.000000	chr11	11723801	11725419	chr11	11270873	11622014	
chr8_82329336_82331284	5.189615	0.000002	0.000043	Il15	-5.185933	0.000810	0.003267	chr4	141733679	141736013	chr4	141636595	141733987	
chr8_102781569_102784308	5.761340	0.000000	0.000000	Cdh11	-6.729272	0.000000	0.000000	chr16	65118621	65121296	chr16	64943752	65122137	
chr11_96341058_96343216	5.337659	0.000001	0.000012	Hoxb3	-6.433657	0.000001	0.000005	chr17	48553538	48555622	chr17	48548869	48582622	Migraine; Insomnia
chr11_96341058_96343216	5.337659	0.000001	0.000012	Hoxb3	-6.433657	0.000001	0.000005	chr17	48553538	48555622	chr17	48548869	48582622	Migraine; Insomnia
chr11_96354206_96355627	5.109476	0.000005	0.000082	Hoxb2	-6.351656	0.000003	0.000022	chr17	48540790	48542479	chr17	48542655	48545031	Cystatin C levels
chr11_96354206_96355627	5.109476	0.000005	0.000082	Hoxb3	-6.433657	0.000001	0.000005	chr17	48540790	48542479	chr17	48548869	48582622	Cystatin C levels
chr12_56506841_56514912	-9.259195	0.000000	0.000000	Nkx2-1	9.134857	0.000000	0.000000	chr14	36491565	36499712	chr14	36516398	36520225	Nonsyndromic orofacial cleft x sex interaction; Nonsyndromic orofacial cleft x sex interaction (2df)
chr13_117409406_117414817	-5.127606	0.000000	0.000000	Emb	-6.021510	0.000004	0.000025				chr5	50396196	50441400	
chr14_66104432_66107303	6.455571	0.000000	0.000000	Ephx2	-6.872942	0.000000	0.000001	chr8	27506903	27511135	chr8	27491001	27544922	Childhood ear infection
chr14_88460573_88463273	5.594736	0.000000	0.000001	Pcdh20	-6.097424	0.000012	0.000070	chr13	61406547	61407676	chr13	61409685	61415522	
chr14_88898125_88900211	-5.263671	0.000002	0.000040	Pcdh20	-6.097424	0.000012	0.000070	chr13	61862674	61862983	chr13	61409685	61415522	
chr15_11692586_11694713	6.046002	0.000000	0.000000	Npr3	-8.356128	0.000000	0.000000	chr5	33023231	33024567	chr5	32710636	32791724	
chr15_11692586_11694713	6.046002	0.000000	0.000000	Npr3	-8.356128	0.000000	0.000000	chr5	33023231	33024567	chr5	32710636	32791724	
chr15_11743634_11745456	6.922288	0.000000	0.000000	Npr3	-8.356128	0.000000	0.000000	chr5	32929090	32932829	chr5	32710636	32791724	
chr15_11743634_11745456	6.922288	0.000000	0.000000	Npr3	-8.356128	0.000000	0.000000	chr5	32929090	32932829	chr5	32710636	32791724	
chr15_11782997_11784930	5.957213	0.000000	0.000000	Npr3	-8.356128	0.000000	0.000000	chr5	32864592	32866245	chr5	32710636	32791724	
chr15_11782997_11784930	5.957213	0.000000	0.000000	Npr3	-8.356128	0.000000	0.000000	chr5	32864592	32866245	chr5	32710636	32791724	
chr18_53465649_53470088	5.761340	0.000000	0.000000	Prdm6	-9.029699	0.000000	0.000000	chr5	123091173	123096087	chr5	123089102	123194266	
chr19_59159949_59161669	-6.094880	0.000000	0.000000	Vax1	7.636277	0.000000	0.000000	chr10	117127894	117129270	chr10	117128520	117138301	
chr19_59163812_59165938	-5.788022	0.000000	0.000000	Vax1	7.636277	0.000000	0.000000	chr10	117131583	117133776	chr10	117128520	117138301	Age at first birth; Nonsyndromic cleft lip with cleft palate
chrX_93281556_93288401	5.093695	0.000000	0.000000	Arx	-5.380834	0.000000	0.000000	chrX	25013967	25020980	chrX	25003695	25015948	
chrY_1009018_1011799	-5.686862	0.000000	0.000001	Eif2s3y	11.645099	0.000000	0.000000	chrX	24054686	24057370				
chrY_1243715_1246316	-5.520433	0.000000	0.000003	Uty	8.369064	0.000000	0.000000	chrY	13478375	13480067	chrY	13248378	13480670	
chrY_1243715_1246316	-5.520433	0.000000	0.000003	Ddx3y	11.525927	0.000000	0.000000	chrY	13478375	13480067	chrY	12903998	12920478	
chrY_1282482_1287504	-5.191646	0.000004	0.000074	Ddx3y	11.525927	0.000000	0.000000	chrY	12903183	12909538	chrY	12903998	12920478	

The columns are: peak name, logfoldchange of peaks, p-value of differential binding analysis, adjusted p-value of differential binding analysis, the paired gene name, score of differential gene analysis, p-value of differential gene analysis, adjusted p-value of differential gene analysis, human chromosome number after ”lifting over” the peak to hg38, human start site after ”lifting over” the peak to hg38, human end site after ”lifting over” the peak to hg38, human chromosome number after ”lifting over” the gene to hg38, human start site after ”lifting over” the gene to hg38, human end site after ”lifting over” the gene to hg38, GWAS information within the peak areas after ”lifting over” the peak to hg38.

4.4 4

Table 4: Motifs Significantly Bounded in Males

Motif Name	Consensus	P-value	Log P-value	q-value (Benjamini)	# of Target Sequences with Motif(of 585)	% of Target Sequences with Motif	# of Background Sequences with Motif(of 48633)	% of Background Sequences with Motif
Nrf2(bZIP)/Lymphoblast-Nrf2-ChIP-Seq(GSE37589)/Homer	HTGCTGAGTCAT	1.000000e-10	-24.030	0.0000	39.0	6.67%	929.4	1.91%
NF-E2(bZIP)/K562-NFE2-ChIP-Seq(GSE31477)/Homer	GATGACTCAGCA	1.000000e-09	-21.300	0.0000	41.0	7.01%	1115.3	2.29%
Bach1(bZIP)/K562-Bach1-ChIP-Seq(GSE31477)/Homer	AWWNTGCTGAGTCAT	1.000000e-08	-19.210	0.0000	39.0	6.67%	1105.7	2.27%
NFE2L2(bZIP)/HepG2-NFE2L2-ChIP-Seq(Encode)/Homer	AWWWTGCTGAGTCAT	1.000000e-07	-17.550	0.0000	48.0	8.21%	1636.5	3.36%
MafK(bZIP)/C2C12-MafK-ChIP-Seq(GSE36030)/Homer	GCTGASTCAGCA	1.000000e-06	-15.170	0.0000	97.0	16.58%	4773.9	9.81%
Ets1-distal(ETS)/CD4+-PolII-ChIP-Seq(Barski_et_al.)/Homer	MACAGGAAGT	1.000000e-05	-13.630	0.0000	116.0	19.83%	6233.9	12.81%
Sox3(HMG)/NPC-Sox3-ChIP-Seq(GSE33059)/Homer	CCWTTGTY	1.000000e-04	-10.180	0.0009	434.0	74.19%	32372.0	66.54%
Bapx1(Homeobox)/VertebralCol-Bapx1-ChIP-Seq(GSE36672)/Homer	TTRAGTGSYK	1.000000e-03	-8.548	0.0043	467.0	79.83%	35725.8	73.43%
LHX9(Homeobox)/Hct116-LHX9.V5-ChIP-Seq(GSE116822)/Homer	NGCTAATTAG	1.000000e-03	-7.670	0.0093	399.0	68.21%	29938.8	61.54%
Nkx2.2(Homeobox)/NPC-Nkx2.2-ChIP-Seq(GSE61673)/Homer	BTBRAGTGSN	1.000000e-03	-7.623	0.0094	419.0	71.62%	31690.5	65.14%
Sox10(HMG)/SciaticNerve-Sox3-ChIP-Seq(GSE35132)/Homer	CCWTTGTYYB	1.000000e-03	-7.534	0.0098	407.0	69.57%	30669.3	63.04%
Lhx2(Homeobox)/HFSC-Lhx2-ChIP-Seq(GSE48068)/Homer	TAATTAGN	1.000000e-03	-7.244	0.0124	343.0	58.63%	25288.8	51.98%
ZNF669(Zf)/HEK293-ZNF669.GFP-ChIP-Seq(GSE58341)/Homer	GARTGGTCATCGCCC	1.000000e-03	-7.173	0.0125	24.0	4.10%	959.3	1.97%
KLF14(Zf)/HEK293-KLF14.GFP-ChIP-Seq(GSE58341)/Homer	RGKGGGCGKGGC	1.000000e-03	-7.144	0.0125	297.0	50.77%	21491.3	44.17%
Sox4(HMG)/proB-Sox4-ChIP-Seq(GSE50066)/Homer	YCTTTGTTCC	1.000000e-03	-7.123	0.0125	255.0	43.59%	18072.7	37.15%
TATA-Box(TBP)/Promoter/Homer	CCTTTTAWAGSC	1.000000e-03	-7.031	0.0130	382.0	65.30%	28658.5	58.91%
Brn1(POU,Homeobox)/NPC-Brn1-ChIP-Seq(GSE35496)/Homer	TATGCWAATBAV	1.000000e-02	-6.282	0.0249	134.0	22.91%	8796.6	18.08%
Sox2(HMG)/mES-Sox2-ChIP-Seq(GSE11431)/Homer	BCCATTGTTC	1.000000e-02	-6.215	0.0259	272.0	46.50%	19720.1	40.53%
RUNX-AML(Runt)/CD4+-PolII-ChIP-Seq(Barski_et_al.)/Homer	GCTGTGGTTW	1.000000e-02	-6.208	0.0259	212.0	36.24%	14887.1	30.60%
Sp5(Zf)/mES-Sp5.Flag-ChIP-Seq(GSE72989)/Homer	RGKGGGCGGAGC	1.000000e-02	-6.201	0.0259	174.0	29.74%	11896.8	24.45%
RUNX1(Runt)/Jurkat-RUNX1-ChIP-Seq(GSE29180)/Homer	AAACCACARM	1.000000e-02	-5.829	0.0323	293.0	50.09%	21565.6	44.33%
Elk4(ETS)/Hela-Elk4-ChIP-Seq(GSE31477)/Homer	NRYTTCCGGY	1.000000e-02	-5.473	0.0447	117.0	20.00%	7700.2	15.83%
CHR(?)/Hela-CellCycle-Expression/Homer	SRGTTTCAAA	1.000000e-02	-5.305	0.0497	225.0	38.46%	16202.3	33.30%
KLF3(Zf)/MEF-Klf3-ChIP-Seq(GSE44748)/Homer	NRGCCCCRCCCHBNN	1.000000e-02	-5.298	0.0497	100.0	17.09%	6462.1	13.28%

The columns are: motif name, consensus of the motif, p-value log p-value q-value (Benjamini), # of target sequences with Motif, % of target sequences with motif, # of background sequences with Motif, % of background sequences with motif.

4.5 5

Table 5: Motifs Significantly Bounded in Females

Motif Name	Consensus	P-value	Log P-value	q-value (Benjamini)	# of Target Sequences with Motif(of 1009)	% of Target Sequences with Motif	# of Background Sequences with Motif(of 48596)	% of Background Sequences with Motif
Chop(bZIP)/MEF-Chop-ChIP-Seq(GSE35681)/Homer	ATTGCATCAT	1.000000e-19	-44.020	0.0000	197.0	19.52%	4851.2	9.98%
Atf4(bZIP)/MEF-Atf4-ChIP-Seq(GSE35681)/Homer	MTGATGCAAT	1.000000e-17	-41.260	0.0000	228.0	22.60%	6117.7	12.59%
ISRE(IRF)/ThioMac-LPS-Expression(GSE23622)/Homer	AGTTTCASTTTC	1.000000e-16	-38.060	0.0000	100.0	9.91%	1872.5	3.85%
IRF3(IRF)/BMDM-Irf3-ChIP-Seq(GSE67343)/Homer	AGTTTCAKTTTC	1.000000e-13	-30.290	0.0000	296.0	29.34%	9507.9	19.57%
IRF2(IRF)/Erythroblas-IRF2-ChIP-Seq(GSE36985)/Homer	GAAASYGAAASY	1.000000e-12	-28.510	0.0000	118.0	11.69%	2788.7	5.74%
IRF8(IRF)/BMDM-IRF8-ChIP-Seq(GSE77884)/Homer	GRAASTGAAAST	1.000000e-12	-28.320	0.0000	255.0	25.27%	7973.4	16.41%
IRF1(IRF)/PBMC-IRF1-ChIP-Seq(GSE43036)/Homer	GAAAGTGAAAGT	1.000000e-09	-21.540	0.0000	147.0	14.57%	4197.4	8.64%
Rbpj1(?)/Panc1-Rbpj1-ChIP-Seq(GSE47459)/Homer	HTTTCCCASG	1.000000e-07	-17.720	0.0000	594.0	58.87%	24396.6	50.20%
CEBP:AP1(bZIP)/ThioMac-CEBPb-ChIP-Seq(GSE21512)/Homer	DRTGTTGCAA	1.000000e-07	-16.770	0.0000	420.0	41.63%	16299.1	33.54%
TEAD1(TEAD)/HepG2-TEAD1-ChIP-Seq(Encode)/Homer	CYRCATTCCA	1.000000e-07	-16.340	0.0000	498.0	49.36%	19987.8	41.13%
Zic3(Zf)/mES-Zic3-ChIP-Seq(GSE37889)/Homer	GGCCYCCTGCTGDGH	1.000000e-06	-15.340	0.0000	189.0	18.73%	6339.3	13.04%
PU.1:IRF8(ETS:IRF)/pDC-Irf8-ChIP-Seq(GSE66899)/Homer	GGAAGTGAAAST	1.000000e-06	-15.240	0.0000	147.0	14.57%	4644.0	9.56%
GABPA(ETS)/Jurkat-GABPa-ChIP-Seq(GSE17954)/Homer	RACCGGAAGT	1.000000e-05	-13.440	0.0000	379.0	37.56%	14873.2	30.61%
Unknown-ESC-element(?)/mES-Nanog-ChIP-Seq(GSE11724)/Homer	CACAGCAGGGGG	1.000000e-05	-12.150	0.0001	223.0	22.10%	8111.5	16.69%
Mef2c(MADS)/GM12878-Mef2c-ChIP-Seq(GSE32465)/Homer	DCYAAAAATAGM	1.000000e-04	-11.440	0.0002	337.0	33.40%	13254.2	27.27%
TEAD3(TEA)/HepG2-TEAD3-ChIP-Seq(Encode)/Homer	TRCATTCCAG	1.000000e-04	-11.430	0.0002	547.0	54.21%	23074.9	47.48%
NF1-halfsite(CTF)/LNCaP-NF1-ChIP-Seq(Unpublished)/Homer	YTGCCAAG	1.000000e-04	-11.190	0.0002	578.0	57.28%	24613.8	50.65%
TEAD(TEA)/Fibroblast-PU.1-ChIP-Seq(Unpublished)/Homer	YCWGGAATGY	1.000000e-04	-10.680	0.0004	383.0	37.96%	15476.2	31.85%
TEAD4(TEA)/Tropoblast-Tead4-ChIP-Seq(GSE37350)/Homer	CCWGGAATGY	1.000000e-04	-10.420	0.0005	409.0	40.54%	16723.6	34.41%
Smad4(MAD)/ESC-SMAD4-ChIP-Seq(GSE29422)/Homer	VBSYGTCTGG	1.000000e-04	-10.240	0.0005	566.0	56.10%	24201.5	49.80%
CEBP:CEBP(bZIP)/MEF-Chop-ChIP-Seq(GSE35681)/Homer	NTNATGCAAYMNNHT-GMAAY	1.000000e-04	-9.796	0.0007	115.0	11.40%	3832.0	7.89%
Pitx1:Ebox(Homeobox,bHLH)/Hindlimb-Pitx1-ChIP-Seq(GSE41591)/Homer	YTAATTRAWWCCA-GATGT	1.000000e-03	-9.188	0.0013	158.0	15.66%	5685.8	11.70%
Dlx3(Homeobox)/Kerainocytes-Dlx3-ChIP-Seq(GSE89884)/Homer	NDGTAATTAC	1.000000e-03	-8.785	0.0019	456.0	45.19%	19220.6	39.55%
PAX6(Paired,Homeobox)/Forebrain-Pax6-ChIP-Seq(GSE66961)/Homer	NGTGTTCAVT-SAAGCGKAAA	1.000000e-03	-8.430	0.0026	70.0	6.94%	2161.0	4.45%
CREB5(bZIP)/LNCaP-CREB5.V5-ChIP-Seq(GSE137775)/Homer	VVATGACGTCAT	1.000000e-03	-8.229	0.0029	206.0	20.42%	7887.2	16.23%
MYB(HTH)/ERMYB-Myb-ChIPSeq(GSE22095)/Homer	GGCVGTTR	1.000000e-03	-7.874	0.0041	659.0	65.31%	29212.3	60.11%
TEAD2(TEA)/Py2T-Tead2-ChIP-Seq(GSE55709)/Homer	CCWGGAATGY	1.000000e-03	-7.798	0.0043	282.0	27.95%	11351.1	23.36%
ETV1(ETS)/GIST48-ETV1-ChIP-Seq(GSE22441)/Homer	AACCGGAAGT	1.000000e-03	-7.698	0.0046	516.0	51.14%	22294.4	45.88%
AMYB(HTH)/Testes-AMYB-ChIP-Seq(GSE44588)/Homer	TGGCAGTTGG	1.000000e-03	-7.598	0.0050	600.0	59.46%	26375.0	54.27%
CArG(MADS)/PUER-Srf-ChIP-Seq(Sullivan_et_al.)/Homer	CCATATATGGNM	1.000000e-03	-7.474	0.0056	183.0	18.14%	6990.5	14.38%
STAT4(Stat)/CD4-Stat4-ChIP-Seq(GSE22104)/Homer	NYTTCCWGGAAR	1.000000e-03	-7.305	0.0063	484.0	47.97%	20850.1	42.91%
NFIL3(bZIP)/HepG2-NFIL3-ChIP-Seq(Encode)/Homer	VTTACGTAAYNNNNN	1.000000e-03	-7.192	0.0068	388.0	38.45%	16347.3	33.64%
HLF(bZIP)/HSC-HLF.Flag-ChIP-Seq(GSE69817)/Homer	RTTATGYAAB	1.000000e-03	-7.106	0.0072	483.0	47.87%	20846.8	42.90%
Six2(Homeobox)/NephronProgenitor-Six2-ChIP-Seq(GSE39837)/Homer	GWAAYHTGAKMC	1.000000e-02	-6.904	0.0087	518.0	51.34%	22564.5	46.43%
Elf4(ETS)/BMDM-Elf4-ChIP-Seq(GSE88699)/Homer	ACTTCCKGKT	1.000000e-02	-6.881	0.0087	421.0	41.72%	17960.0	36.96%
Zic(Zf)/Cerebellum-ZIC1.2-ChIP-Seq(GSE60731)/Homer	CCTGCTGAGH	1.000000e-02	-6.835	0.0089	309.0	30.62%	12762.1	26.26%
NFkB-p65-Rel(RHD)/ThioMac-LPS-Expression(GSE23622)/Homer	GGAAATTCCC	1.000000e-02	-6.800	0.0089	34.0	3.37%	917.9	1.89%
EBF1(EBF)/Near-E2A-ChIP-Seq(GSE21512)/Homer	GTCCCCWGGGGA	1.000000e-02	-6.654	0.0101	310.0	30.72%	12844.1	26.43%
Mef2a(MADS)/HL1-Mef2a.biotin-ChIP-Seq(GSE21529)/Homer	CYAAAAATAG	1.000000e-02	-6.569	0.0108	278.0	27.55%	11396.8	23.45%
PU.1-IRF(ETS:IRF)/Bcell-PU.1-ChIP-Seq(GSE21512)/Homer	MGGAAGTGAAAC	1.000000e-02	-6.543	0.0109	599.0	59.37%	26563.8	54.66%
IRF4(IRF)/GM12878-IRF4-ChIP-Seq(GSE32465)/Homer	ACTGAAACCA	1.000000e-02	-6.485	0.0114	266.0	26.36%	10866.8	22.36%
Zic2(Zf)/ESC-Zic2-ChIP-Seq(SRP197560)/Homer	CHCAGCRGGRGG	1.000000e-02	-6.257	0.0138	140.0	13.88%	5296.3	10.90%
Elk1(ETS)/Hela-Elk1-ChIP-Seq(GSE31477)/Homer	HACTTCCGGY	1.000000e-02	-6.215	0.0142	183.0	18.14%	7187.3	14.79%
Smad2(MAD)/ES-SMAD2-ChIP-Seq(GSE29422)/Homer	CTGTCTGG	1.000000e-02	-6.069	0.0162	545.0	54.01%	24059.7	49.51%
Mef2d(MADS)/Retina-Mef2d-ChIP-Seq(GSE61391)/Homer	GCTATTTTTAGC	1.000000e-02	-6.020	0.0167	136.0	13.48%	5157.9	10.61%
c-Jun-CRE(bZIP)/K562-cJun-ChIP-Seq(GSE31477)/Homer	ATGACGTCATCY	1.000000e-02	-6.000	0.0168	133.0	13.18%	5030.2	10.35%
NFAT:AP1(RHD,bZIP)/Jurkat-NFATC1-ChIP-Seq(Jolma_et_al.)/Homer	SARTGGAAAAWRT-GAGTCAB	1.000000e-02	-5.920	0.0179	110.0	10.90%	4053.8	8.34%
Prop1(Homeobox)/GHFT1-PROP1.biotin-ChIP-Seq(GSE77302)/Homer	NTAATBNAATTA	1.000000e-02	-5.844	0.0189	446.0	44.20%	19380.0	39.88%
IRF:BATF(IRF:bZIP)/pDC-Irf8-ChIP-Seq(GSE66899)/Homer	CTTTCANTATGACTV	1.000000e-02	-5.825	0.0189	98.0	9.71%	3556.1	7.32%
PAX3:FKHR-fusion(Paired,Homeobox)/Rh4-PAX3:FKHR-ChIP-Seq(GSE19063)/Homer	ACCRTGACTAATTNN	1.000000e-02	-5.789	0.0192	152.0	15.06%	5890.0	12.12%
STAT6(Stat)/Macrophage-Stat6-ChIP-Seq(GSE38377)/Homer	TTCCKNAGAA	1.000000e-02	-5.721	0.0203	305.0	30.23%	12810.3	26.36%
Hoxd11(Homeobox)/ChickenMSG-Hoxd11.Flag-ChIP-Seq(GSE86088)/Homer	VGCCATAAAA	1.000000e-02	-5.700	0.0204	895.0	88.70%	41669.0	85.75%
STAT6(Stat)/CD4-Stat6-ChIP-Seq(GSE22104)/Homer	ABTTCYYRRGAA	1.000000e-02	-5.632	0.0216	308.0	30.53%	12968.7	26.69%
DLX1(Homeobox)/BasalGanglia-Dlx1-ChIP-seq(GSE124936)/Homer	NSNNTAATTA	1.000000e-02	-5.582	0.0224	684.0	67.79%	30969.0	63.73%
ETS:E-box(ETS,bHLH)/HPC7-Scl-ChIP-Seq(GSE22178)/Homer	AGGAARCAGCTG	1.000000e-02	-5.399	0.0265	50.0	4.96%	1625.2	3.34%
PU.1(ETS)/ThioMac-PU.1-ChIP-Seq(GSE21512)/Homer	AGAGGAAGTG	1.000000e-02	-5.341	0.0277	240.0	23.79%	9912.9	20.40%
Stat3(Stat)/mES-Stat3-ChIP-Seq(GSE11431)/Homer	CTTCCGGGAA	1.000000e-02	-5.293	0.0287	227.0	22.50%	9331.1	19.20%
EHF(ETS)/LoVo-EHF-ChIP-Seq(GSE49402)/Homer	AVCAGGAAGT	1.000000e-02	-4.905	0.0413	543.0	53.82%	24265.3	49.93%
NFkB-p65(RHD)/GM12787-p65-ChIP-Seq(GSE19485)/Homer	WGGGGATTTCCC	1.000000e-02	-4.861	0.0426	206.0	20.42%	8468.5	17.43%
Brn2(POU,Homeobox)/NPC-Brn2-ChIP-Seq(GSE35496)/Homer	ATGAATATTC	1.000000e-02	-4.829	0.0434	86.0	8.52%	3175.7	6.53%
BMYB(HTH)/Hela-BMYB-ChIP-Seq(GSE27030)/Homer	NHAACBGYYV	1.000000e-02	-4.779	0.0451	591.0	58.57%	26624.5	54.79%

The columns are: motif name, consensus of the motif, p-value log p-value q-value (Benjamini), # of target sequences with Motif, % of target sequences with motif, # of background sequences with Motif, % of background sequences with motif.

4.6 6

Table 6: Differentiated Results for Yeast Data

Peak	group	logfoldchanges	pvalues	footprint_start	footprint_end	Gene Name1	log2_ratio_GCR2_1	log2_ratio_TYE7_1	M_value_1	p_value_1	Gene Name2	log2_ratio_GCR2_1	log2_ratio_TYE7_1	M_value_2	p_value_2
chrI_71058_71450	TYE7	2.784531	1.953979e-07	71216	71400	CDC19	1.298305		-1.2954679	7.55187e-30	CYC3				
chrII_613940_614505	TYE7	5.382557	4.503557e-38	614133	614356	PGI1	1.168496	1.236167			TAF5				
chrII_614793_615361	TYE7	4.959104	1.137108e-18	615046	615270	TAF5					PGI1	1.168496	1.236167		
chrIII_137273_137488	TYE7	1.820730	3.459797e-02	137342	137398	PGK1	2.180786	2.593547			ADP1				
chrIX_254382_254905	TYE7	1.666128	1.254026e-11	254516	254741	RPL34B					VHR1				
chrV_498142_498323	TYE7	1.727651	5.307259e-02	498142	498323	SPT2					RAD4				
chrV_544670_545587	TYE7	3.728325	9.272983e-33	545118	545407	ECM32					BMH1				
chrVII_883857_884257	TYE7	3.606330	1.927126e-70	884080	884176	TDH3	1.070149	1.794388			PDX1				
chrVII_999899_1001006	TYE7	2.295043	6.436514e-40	1000238	1000631	ENO1	2.635987	2.795574	-1.424761	0.0	PUP2				
chrVIII_450860_451208	TYE7	6.446487	2.321509e-28	450990	451068	SPC97					ENO2	2.270574	1.996884	-1.570186	7.988330e-14
chrVIII_549242_549430	TYE7	0.313615	6.755711e-01	549320	549377	PHO12					IMD2			1.627560	0.000000e+00
chrX_454688_455338	TYE7	3.949230	2.108841e-51	454946	455020	TDH2	2.091124	2.408665			MET3				
chrXI_164749_164897	TYE7	3.724329	4.839595e-04	164749	164897	GPM1	2.892607	2.502229	-2.781464	0.0	MCR1				
chrXI_327288_327611	TYE7	1.728583	2.634327e-07	327411	327481	FBA1	1.682776	1.640464			MPE1				
chrXII_220643_220821	TYE7	2.724692	5.932963e-02	220643	220821	PAU23					COX12				
chrXII_234178_234860	TYE7	1.181715	2.260771e-07	234387	234786	PDC1					STU2				
chrXII_650657_651279	TYE7	0.842179	6.521443e-04	650826	651158	HAP1					NDL1				
chrXIII_674979_675319	TYE7	2.503483	5.556749e-18	674979	675319	PFK2	1.045008	1.002961			HFA1				
chrXV_160747_161147	TYE7	3.246710	4.388851e-07	160917	161116	ADH1	2.309545	2.368507			PHM7				
chrXV_970959_971080	TYE7	0.576370	5.822512e-01	970959	971080	RPA190					TYE7		5.472818	1.326491	9.292210e-29
chrXVI_411349_411915	TYE7	4.133628	5.321212e-19	411528	411713	GPI2					GCR1	1.986930	1.801998		
chrXVI_412048_412354	TYE7	13.863855	2.628075e-17	412163	412233	GCR1	1.986930	1.801998			GPI2				
chrI_229758_229966	TYE7_gcr2ko	1.347771	2.715033e-03	229803	229906	PHO11					FLO1				
chrI_68120_68335	TYE7_gcr2ko	0.914803	1.150768e-01	68246	68324	CYC3					CLN3				
chrII_221245_221577	TYE7_gcr2ko	2.133472	9.539305e-11	221449	221557	PDR3					LDB7				
chrII_260413_260575	TYE7_gcr2ko	1.123237	8.061584e-02	260466	260535	IPP1					HHT1				
chrII_29937_30481	TYE7_gcr2ko	0.960776	5.032881e-03	30129	30410	ECM21					SFT2				
chrII_455444_455788	TYE7_gcr2ko	4.493130	1.004659e-05	455557	455688	AIM3					IML3				
chrII_533304_533666	TYE7_gcr2ko	3.800928	2.132744e-11	533329	533500	ADH5		1.361025	1.538598	0.0	SUP45				
chrIII_50464_50773	TYE7_gcr2ko	0.745053	5.691017e-02	50528	50645	GLK1		1.208995			PDI1				
chrIII_68547_68827	TYE7_gcr2ko	1.137314	3.027997e-03	68634	68742	BIK1					HIS4				
chrIV_1270863_1270994	TYE7_gcr2ko	12.035796	2.517398e-06	1270863	1270994	URH1					HPT1				
chrIV_1476711_1477076	TYE7_gcr2ko	1.078203	9.265489e-02	1476832	1476955	GRH1					EMI2				
chrIV_215708_216063	TYE7_gcr2ko	3.480023	3.447024e-20	215806	215959	RGT2					ARF2				
chrIV_314944_315311	TYE7_gcr2ko	2.051902	2.535867e-04	315108	315190	MDH3					MRK1				
chrIV_366331_366464	TYE7_gcr2ko	1.591946	7.600754e-02	366331	366464	STP4					KNH1				
chrIV_413655_413831	TYE7_gcr2ko	2.006380	3.227108e-02	413655	413831	GPM2		1.482985	1.001564	0.0	GPD1				
chrIV_803215_803664	TYE7_gcr2ko	2.762052	1.002192e-06	803360	803582	SEC7					HSP42				
chrIV_927099_927255	TYE7_gcr2ko	11.550507	1.005197e-04	927099	927255	COX20					HEM1				
chrIV_955079_955456	TYE7_gcr2ko	1.158595	1.677631e-03	955191	955295	TRS23					VHS1				
chrIV_981412_981615	TYE7_gcr2ko	1.007892	8.040092e-02	981480	981562	EXG2					SWM1				
chrIV_981781_982057	TYE7_gcr2ko	0.899775	4.807948e-05	981911	981978	EXG2					SWM1				
chrV_291305_291670	TYE7_gcr2ko	2.236300	4.226114e-05	291407	291609	RGI1					MOT2				
chrV_311589_311898	TYE7_gcr2ko	2.399778	7.190005e-06	311698	311856	PTP3					YOS1				
chrVII_14054_15155	TYE7_gcr2ko	0.245971	6.077501e-03	14500	14837	MNT2					ADH4				
chrVII_567315_567563	TYE7_gcr2ko	0.123640	8.551552e-01	567380	567492	ORM1					ACB1				
chrVII_574203_574357	TYE7_gcr2ko	1.592754	1.697728e-03	574203	574357	KSS1					BUD9				
chrVII_625218_625597	TYE7_gcr2ko	3.177404	7.027647e-14	625385	625509	ART5					ROM1				
chrVIII_548599_548842	TYE7_gcr2ko	1.415932	5.508430e-03	548670	548761	PHO12					IMD2			1.627560	0.000000e+00
chrX_337351_338085	TYE7_gcr2ko	0.883445	3.808735e-18	337611	337820	TDH1	1.968924	3.133345	-1.115719	0.000004	PEP8				
chrXI_308326_308525	TYE7_gcr2ko	0.914235	4.978495e-01	308326	308525	NUP100					YNK1				
chrXI_381717_381964	TYE7_gcr2ko	0.330000	6.645344e-01	381793	381878	IXR1					MAE1				
chrXI_384529_384715	TYE7_gcr2ko	1.399387	1.283071e-01	384529	384715	MAE1					TFA1				
chrXIII_184375_184617	TYE7_gcr2ko	0.592995	2.671287e-01	184439	184549	PRP39					PRM6				
chrXIII_361934_362209	TYE7_gcr2ko	2.134604	8.382418e-21	362045	362141	NUP116					CSM3				
chrXIII_362321_362629	TYE7_gcr2ko	1.688819	3.481021e-13	362400	362485	NUP116					CSM3				
chrXIII_773716_774065	TYE7_gcr2ko	2.040118	1.806886e-05	773809	773951	GTO3					HOR7				
chrXIV_101357_102062	TYE7_gcr2ko	1.487317	5.251183e-18	101589	101913	MRPL10					WSC2				
chrXIV_301779_302150	TYE7_gcr2ko	4.589684	3.215739e-11	301870	302016	RPS3					RHO5				
chrXIV_519627_520135	TYE7_gcr2ko	0.126028	5.065225e-01	519788	520004	POR1					OCA2				
chrXIV_567177_567791	TYE7_gcr2ko	0.943612	1.085177e-05	567298	567657	NCE103					SIW14				
chrXV_117912_118160	TYE7_gcr2ko	0.136291	6.403453e-01	117981	118082	NDJ1		1.051371	1.205167	0.0	WSC3				
chrXV_216457_216739	TYE7_gcr2ko	0.785741	6.482490e-03	216578	216655	MAM3					GPD2				
chrXV_304395_304786	TYE7_gcr2ko	3.399200	5.453564e-10	304540	304745	HTZ1					PLB3				
chrXV_670788_671140	TYE7_gcr2ko	1.762657	2.358717e-04	670919	671048	GAC1					SYC1				
chrXV_709169_709425	TYE7_gcr2ko	1.214541	3.026343e-05	709267	709349	PEX27					TOA1				
chrXV_987325_988055	TYE7_gcr2ko	0.567374	2.918835e-03	987523	987900	PUT4					PYK2				
chrXV_988153_988367	TYE7_gcr2ko	1.078203	9.265489e-02	988214	988319	PUT4					CIN1				
chrXVI_855654_856341	TYE7_gcr2ko	1.112779	2.739031e-23	855800	856103	KRE6					GPH1				

The columns are: peak name, the condition in which the peak is differentiated bound, logfoldchange of binding (positive means more tightly bound in WT yeast, negative means more tightly bound in gcr2delta yeast), p-value of differential binding analysis, the starting position of footprint analysis, the ending position of

footprint analysis, the name of the closest gene1, log2 ratio of the knockdown *gcr2*Δ for gene1 (Hackett et al., 2020), log2 ratio of the knockdown Tye7p for gene1 (Hackett et al., 2020), the M value (log2 expression ratio) of the *gcr2*Δ knockdown data for gene1 (Kemmeren et al., 2014), the p-value of the *gcr2*Δ knockdown data for gene 1 (Kemmeren et al., 2014), the name of the second cloest gene2, log2 ratio of the knockdown *gcr2*Δ for gene2 (Hackett et al., 2020), log2 ratio of the knockdown Tye7p for gene2 (Hackett et al., 2020), the M value (log2 expression ratio) of the *gcr2*Δ knockdown data for gene2 (Kemmeren et al., 2014), the p-value of the *gcr2*Δ knockdown data for gene 2 (Kemmeren et al., 2014).

Table 7: Barcoded PB_SRT_Puro (pRM 1892)

Name	Sequence	Purification	Note
SMART_dT18VN	AAGCAGTGGTATCAACGCAGAGTACGTTTTTTTTTTTTTTTTTTTTTTTTTTTTTTVN	Standard desalt	RT primer for bulk RNA calling card recovery
SMART	AAGCAGTGGTATCAACGCAGAGT	Standard desalt	PCR primer for bulk RNA calling card amplification
SRT_PAC_F1	CAACCTCCCCTTCTACGAGC	Standard desalt	Puromycin marker in SRT
Raff_ACTB_F	CCTCGCCTTTGCCGATCCG	Standard desalt	Human ACTB primer (for RT control)
Raff_ACTB_R	GGATCTTCATGAGGTAGTCAGTCAGGTCC	Standard desalt	Human ACTB primer (for RT control)
OMPBACGIndex2	AATGATACGGCGACCACCGAGATCTACAC[index2]ACACTCTTTCCCTACACGACGCTCTTCCGATCTACGCGTCAATTTTACGCAGACTATCTTT	Standard desalt	For use with piggyBac SRTs with indexes for Novaseq sequencing
OMPBCTAIndex2	AATGATACGGCGACCACCGAGATCTACAC[index2]ACACTCTTTCCCTACACGACGCTCTTCCGATCTCTACGTCAATTTTACGCAGACTATCTTT	Standard desalt	
N7 index1 prime	CAAGCAGAAGACGGCATACGAGAT[index1]GTCTCGTGGGCTCGG	Standard desalt	Unique index1 identifies each bulk RNA calling card library for Novaseq sequencing

Table 8: Summary of bulk calling cards RNA seq experiments

Cell line	Construct	Replictes
K562	HyPBase(pRM 1011)	10
K562	HyPBase_centrip(pRM 1114)	10
K562	Barcoded PB_SRT_Puro(pRM 1892)	

4.7 Recommended model/parameters settings for peaks calling or differential peaks analysis

Table 9: Recommended model/parameters settings for peaks calling of different situations

SITUATION	METHOD	SETTINGS
Single-cell calling cards data without background	CCcaller	maxbetween = 1000-2000; pvalue_adj_cutoff = 0.001-0.05; lam_win_size = 1000000; pseudocounts = 0.1-1*.
Single-cell calling cards data with background	MACCs	window_size = 1000-2000; step_size = 500-800; pvalue_cutoffTTAA = 0.001-0.05; pvalue_cutoffbg = 0.1; lam_win_size = 1000000; pseudocounts = 0.1-1*.
Bulk calling cards data without background	CCcaller	maxbetween = 800-1200; pvalue_adj_cutoff = 0.0001-0.01; lam_win_size = 1000000; pseudocounts = 0.1-20*.
Bulk calling cards data with background	MACCs	window_size = 1000-1200; step_size = 500-800; pvalue_cutoffTTAA = 0.0001-0.01; pvalue_cutoffbg = 0.001-0.1; lam_win_size = 1000000; pseudocounts = 0.1-20 *.
Yeast calling cards data	MACCs	window_size = 100-200; step_size = 30-80; pvalue_cutoff = 0.0001-0.01; lam_win_size = 1000000; pseudocounts = 1*.

*The setting of pseudocounts is largely influenced by library size. Normally, it can be adjusted to $10^{-6} - 10^{-5} \times$ the number of insertions.

In general, we recommend using CCcaller for calling Brd4 (undirected) peaks or for calling TF peaks when no background is available or when the TF strongly redirects the HyPBase and no background is needed. We recommend MACCs for calling TF peaks when there is a Brd4 background available.
For differential analysis, the Fisher exact test is the primary method for calling differentially bound peaks while the binomial test is primarily included as an alternative method and for backward compatibility with previous peak calling methods.

References

Andrilenas, K. K., Ramlall, V., Kurland, J., Leung, B., Harbaugh, A. G., & Siggers, T. (2018). Dna-binding landscape of irf3, irf5 and irf7 dimers: implications for dimer-specific gene regulation. *Nucleic acids research*, *46*(5), 2509–2520.

Beisel, C., Jordan-Paiz, A., Köllmann, S., Ahrenstorf, A. E., Padoan, B., Barkhausen, T., … Altfeld, M. (2023). Sex differences in the percentage of irf5 positive b cells are associated with higher production of tnf-α in women in response to tlr9 in humans. *Biology of Sex Differences*, *14*(1), 11.

Bredikhin, D., Kats, I., & Stegle, O. (2022). Muon: multimodal omics analysis framework. *Genome Biology*, *23*(1), 1–12.

Consortium, E. P., et al. (2012). An integrated encyclopedia of dna elements in the human genome. *Nature*, *489*(7414), 57.

Dong, X., Hu, X., Bao, Y., Li, G., Yang, X.-d., Slauch, J. M., & Chen, L.-F. (2021). Brd4 regulates nlrc4 inflammasome activation by facilitating irf8-mediated transcription of naips. *Journal of Cell Biology*, *220*(3).

Fatima, M., Zhang, X., Lin, J., Zhou, P., Zhou, D., & Ming, R. (2020). Expression profiling of mads-box gene family revealed its role in vegetative development and stem ripening in s. spontaneum. *Scientific reports*, *10*(1), 20536.

Gogol-Döring, A., Ammar, I., Gupta, S., Bunse, M., Miskey, C., Chen, W., … Ivics, Z. (2016). Genome-wide profiling reveals remarkable parallels between insertion site selection properties of the mlv retrovirus and the piggybac transposon in primary human cd4+ t cells. *Molecular Therapy*, *24*(3), 592–606.

Grant, O. A., Wang, Y., Kumari, M., Zabet, N. R., & Schalkwyk, L. (2022). Characterising sex differences of autosomal dna methylation in whole blood using the illumina epic array. *Clinical epigenetics*, *14*(1), 62.

Griesbeck, M., Ziegler, S., Laffont, S., Smith, N., Chauveau, L., Tomezsko, P., … others (2015). Sex differences in plasmacytoid dendritic cell levels of irf5 drive higher ifn-α production in women. *The Journal of Immunology*, *195*(11), 5327–5336.

Hackett, S. R., Baltz, E. A., Coram, M., Wranik, B. J., Kim, G., Baker, A., … McIsaac, R. S. (2020). Learning causal networks using inducible transcription factors and transcriptome-wide time series. *Molecular systems biology*, *16*(3), e9174.

Heinz, S., Benner, C., Spann, N., Bertolino, E., Lin, Y. C., Laslo, P., … Glass, C. K. (2010). Simple combinations of lineage-determining transcription factors prime cis-regulatory elements required for macrophage and b cell identities. *Molecular cell*, *38*(4), 576–589.

Kemmeren, P., Sameith, K., Van De Pasch, L. A., Benschop, J. J., Lenstra, T. L., Margaritis, T., … others (2014). Large-scale genetic perturbations reveal regulatory networks and an abundance of gene-specific repressors. *Cell*, *157*(3), 740–752.

Kent, W. J., Zweig, A. S., Barber, G., Hinrichs, A. S., & Karolchik, D. (2010). Bigwig and bigbed: enabling browsing of large distributed datasets. *Bioinformatics*, *26*(17), 2204–2207.

Kfoury, N., Qi, Z., Prager, B. C., Wilkinson, M. N., Broestl, L., Berrett, K. C., … others (2021). Brd4-bound enhancers drive cell-intrinsic sex differences in glioblastoma. *Proceedings of the National Academy of Sciences*, *118*(16), e2017148118.

Li, D., Hsu, S., Purushotham, D., Sears, R. L., & Wang, T. (2019). Washu epigenome browser update 2019. *Nucleic acids research*, *47*(W1), W158–W165.

Li, D., Purushotham, D., Harrison, J. K., Hsu, S., Zhuo, X., Fan, C., … others (2022). Washu epigenome browser update 2022. *Nucleic acids research*, *50*(W1), W774–W781.

McLachlan, G. J., & Krishnan, T. (2007). *The em algorithm and extensions*. John Wiley & Sons.

Moudgil, A., Li, D., Hsu, S., Purushotham, D., Wang, T., & Mitra, R. D. (2021). The qbed track: a novel genome browser visualization for point processes. *Bioinformatics*, *37*(8), 1168–1170.

Moudgil, A., Wilkinson, M. N., Chen, X., He, J., Cammack, A. J., Vasek, M. J., … others (2020). Self-reporting transposons enable simultaneous readout of gene expression and transcription factor binding in single cells. *Cell*, *182*(4), 992–1008.

Okada, Y., Nobori, H., Shimizu, M., Watanabe, M., Yonekura, M., Nakai, T., … others (2011). Multiple ets family proteins regulate pf4 gene expression by binding to the same ets binding site. *PloS one*, *6*(9), e24837.

Quinlan, A. R., & Hall, I. M. (2010). Bedtools: a flexible suite of utilities for comparing genomic features. *Bioinformatics*, *26*(6), 841–842.

Shively, C. A., Liu, J., Chen, X., Loell, K., & Mitra, R. D. (2019). Homotypic cooperativity and collective binding are determinants of bhlh specificity and function. *Proceedings of the National Academy of Sciences*, *116*(32), 16143–16152.

Thissen, D., Steinberg, L., & Kuang, D. (2002). Quick and easy implementation of the benjamini-hochberg procedure for controlling the false positive rate in multiple comparisons. *Journal of educational and behavioral statistics*, *27*(1), 77–83.

Weiss, M. A. (2005). Molecular mechanisms of male sex determination: the enigma of sry. *DNA Conformation and Transcription*, 1–15.

Wolf, F. A., Angerer, P., & Theis, F. J. (2018). Scanpy: large-scale single-cell gene expression data analysis. *Genome biology*, *19*(1), 1–5.

Zhang, J., Lee, D., Dhiman, V., Jiang, P., Xu, J., McGillivray, P., … others (2020). An integrative encode resource for cancer genomics. *Nature communications*, *11*(1), 3696.

Zhang, J., Liu, J., Lee, D., Lou, S., Chen, Z., Gürsoy, G., & Gerstein, M. (2020). Diner: a differential graphical model for analysis of co-regulation network rewiring. *BMC bioinformatics*, *21*(1), 1–15.

Zhang, X., Wang, X., Pan, L., Guo, W., Li, Y., & Wang, W. (2023). Genome-wide identification and expression analysis of mads-box transcription factors reveal their involvement in sex determination of hardy rubber tree (eucommia ulmoides oliv.). *Frontiers in Genetics*, *14*, 1138703.

Zhang, Y., Liu, T., Meyer, C. A., Eeckhoute, J., Johnson, D. S., Bernstein, B. E., … others (2008). Model-based analysis of chip-seq (macs). *Genome biology*, *9*(9), 1–9.
